# A new insight into aggregation of oncolytic adenovirus Ad5-delta-24-RGD during CsCl gradient ultracentrifugation

**DOI:** 10.1038/s41598-021-94573-y

**Published:** 2021-08-09

**Authors:** Aleksei A. Stepanenko, Anastasiia O. Sosnovtseva, Marat P. Valikhov, Vladimir P. Chekhonin

**Affiliations:** 1 Department of Fundamental and Applied Neurobiology, V.P. Serbsky National Medical Research Center of Psychiatry and Narcology, The Ministry of Health of the Russian Federation, Kropotkinsky Lane 23, Moscow, 119034 Russia; 2 Department of Medical Nanobiotechnology, Institute of Translational Medicine, N.I. Pirogov Russian National Research Medical University, The Ministry of Health of the Russian Federation, Ostrovitianov Str. 1, Moscow, 117997 Russia

**Keywords:** Biochemistry, Biological techniques, Biotechnology, Cancer, Genetics, Molecular biology, Oncology

## Abstract

Two-cycle cesium chloride (2 × CsCl) gradient ultracentrifugation is a conventional approach for purifying recombinant adenoviruses (rAds) for research purposes (gene therapy, vaccines, and oncolytic vectors). However, rAds containing the RGD-4C peptide in the HI loop of the fiber knob domain tend to aggregate during 2 × CsCl gradient ultracentrifugation resulting in a low infectious titer yield or even purification failure. An iodixanol-based purification method preventing aggregation of the RGD4C-modified rAds has been proposed. However, the reason explaining aggregation of the RGD4C-modified rAds during 2 × CsCl but not iodixanol gradient ultracentrifugation has not been revealed. In the present study, we showed that rAds with the RGD-4C peptide in the HI loop but not at the C-terminus of the fiber knob domain were prone to aggregate during 2 × CsCl but not iodixanol gradient ultracentrifugation. The cysteine residues with free thiol groups after the RGD motif within the inserted RGD-4C peptide were responsible for formation of the interparticle disulfide bonds under atmospheric oxygen and aggregation of Ad5-delta-24-RGD4C-based rAds during 2 × CsCl gradient ultracentrifugation, which could be prevented using iodixanol gradient ultracentrifugation, most likely due to antioxidant properties of iodixanol. A cysteine-to-glycine substitution of the cysteine residues with free thiol groups (RGD-2C2G) prevented aggregation during 2 × CsCl gradient purification but in coxsackie and adenovirus receptor (CAR)-low/negative cancer cell lines of human and rodent origin, this reduced cytolytic efficacy to the levels observed for a fiber non-modified control vector. However, both Ad5-delta-24-RGD4C and Ad5-delta-24-RGD2C2G were equally effective in the murine immunocompetent CT-2A glioma model due to a primary role of antitumor immune responses in the therapeutic efficacy of oncolytic virotherapy.

## Introduction

Human adenovirus 5 (Ad5)-based oncolytic recombinant viruses (rAds) have been the most commonly used across many cancer types in clinical trials. Ad5-delta-24-RGD (DNX-2401) is a replication-competent, infectivity-enhanced rAd^1^, which is currently under investigation in a number of phase I/II clinical trials in patients with high-grade glioma (WHO grade III-IV malignant brain tumors)^2–4^. In a pivotal phase I trial of Ad5-delta-24-RGD in patients with recurrent glioma, five patients survived > 3 years and three patients showed > 3 years of progression-free survival from twenty five patients that received a single intratumoral injection dose^5^. Ad5-delta-24-RGD contains two genetic modifications. A first modification is the deletion of a 24-base pair sequence in the conservative region 2 (CR2) domain of the E1A protein (delta-24, or *E1A*Δ24), disrupting a binding site to the retinoblastoma protein (pRb) that prevents effective replication of rAds in normal quiescent or G_1_-arrested cells with intact pRb^6,7^. A second modification is the incorporation of the arginine-glycine-aspartate (RGD)-motif containing, αV-integrin binding peptide RGD-4C (CDC**RGD**CFC) in the HI loop of the fiber knob domain^8^. Wild type Ad5 transduces host cells by attaching via the fiber knob domain to coxsackievirus and adenovirus receptor (CAR) and internalizing by endocytosis through the interaction of the conserved RGD sequences of its penton base proteins with αVβ3/αVβ5 integrins. However, expression of CAR is frequently barely detectable in primary tumor cells reducing Ad5 infection^9–13^. Ad5 tropism modification by inserting the RGD-4C peptide in the HI loop of the fiber knob domain enhanced CAR-independent transduction of a wide range of tumor cells of different tissue origin, including glioma^1,14,15^.

Two-cycle cesium chloride (2 × CsCl) gradient ultracentrifugation is a conventional mid-scale approach for purifying rAds for research purposes, with Ad5-delta-24-RGD-based rAds being not an exception (e.g.^16–18^). However, it was observed that rAds containing the RGD-4C peptide in the HI loop of the fiber knob domain tended to aggregate during 2 × CsCl gradient ultracentrifugation, especially in the second round of ultracentrifugation, forming floccules without a sharp band, which resulted in a low infectious titer yield or even purification failure^19^. It was hypothesized that altered interactions between virus-virus or virus-cellular proteins may be responsible for aggregation of the RGD4C-modified rAds during 2 × CsCl banding^19^. An alternative purification method preventing aggregation of rAds with the RGD-4C peptide in the HI loop of the fiber knob domain has been proposed, which is based on iodixanol (a nonionic hydrophilic radiographic contrast agent) discontinuous density ultracentrifugation followed by size exclusion column chromatography^19^. However, since then, the actual reason explaining aggregation of rAds with the RGD-4C peptide in the HI loop of the fiber knob domain during 2 × CsCl but not iodixanol gradient ultracentrifugation has never been revealed. In this study, we provided a mechanistic explanation on this long-standing issue.

## Results

### Recombinant adenoviruses with the RGD-4C peptide in the HI loop of the fiber knob domain but not at the C-terminus of the serotype chimeric fibers aggregate during 2 × CsCl gradient ultracentrifugation

**Figure 1:**
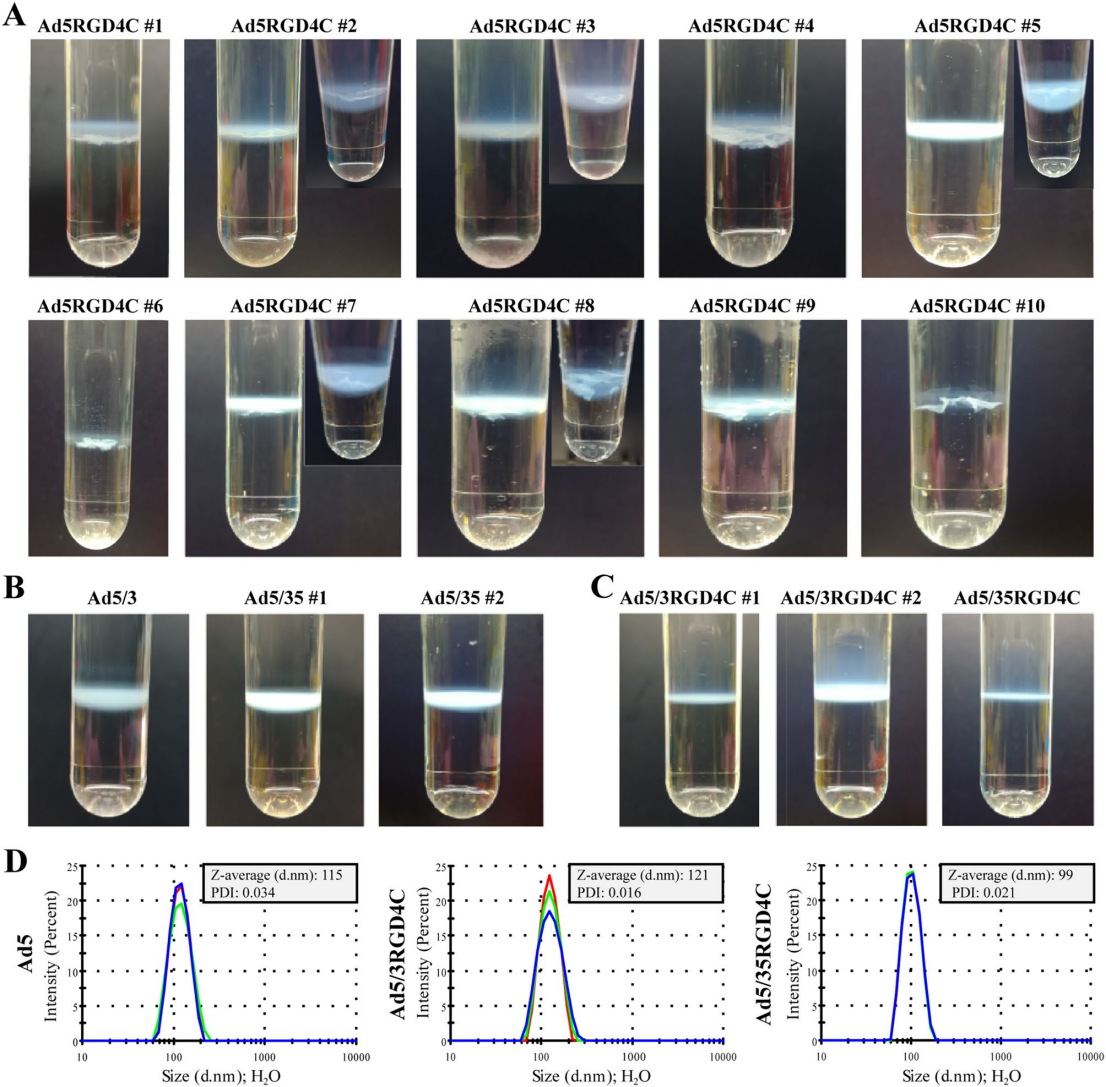
Recombinant adenoviruses with the RGD4C peptide inserted in the HI loop of the fiber knob domain form visible macroscopic and/or invisible microscopic aggregates during conventional two-cycle CsCl gradient purification. (**A**) Representative pictures of ten independent preparations of Ad5-delta-24-RGD4C-based rAds aggregated during 2 × CsCl gradient purification, especially in the second round of ultracentrifugation, forming floccules without a sharp band. (**B**) No macroscopic aggregation was observed for fiber-unmodified Ad5-delta-24 (two preparations) or fiber-chimeric Ad5/3-delta-24 (four preparations) and Ad5/35-delta-24 (six preparations). Representative pictures are shown. (**C**) No macroscopic aggregation was observed for complex fiber-chimeric Ad5/3-delta-24-RGD4C (two independent preparations) and Ad5/35-delta-24-RGD4C with the RGD-4C peptide fused to the C-terminus of the chimeric fiber via a glycine-serine 3 × (G4S) linker. (**D**) The absence of microscopic aggregation for all macroscopically non-aggregated rAds was confirmed using photon correlation spectroscopy, which measures size distributions by intensity, the intensity weighted mean hydrodynamic sizes (Z-averages, d.nm) and polydispersity indices (PDI) of nanoparticles in suspension. Representative measurements are shown. The measurements were carried out at a rAd concentration of 3 × 10^10^ vp in 1 ml total volume in deionized water immediately after viral aliquot sample thawing. Each color curve represents an instrumental individual measurement record.

Purifying a panel of genetically modified oncolytic adenoviruses^20,21^ based on Ad5-delta-24-RGD4C with the RGD-4C peptide inserted in the HI loop of the fiber knob domain, we frequently observed visible macroscopic aggregation with varying degrees during 2 × CsCl gradient ultracentrifugation (Fig. 1A). No macroscopic aggregation was observed for fiber-unmodified Ad5-delta-24 or fiber-chimeric Ad5/3-delta-24 and Ad5/35-delta-24, which fibers consisted of the knob domains (and shaft for 5/35) derived from human Ad3 and Ad35 serotypes, respectively, for retargeting to desmoglein 2 (DSG2) and CD46 receptors (Fig. 1B). Moreover, no macroscopic aggregation during 2 × CsCl gradient ultracentrifugation was also observed for complex fiber-chimeric Ad5/3-delta-24-RGD4C and Ad5/35-delta-24-RGD4C, which fibers combined serotype chimerism with the RGD-4C peptide fused to the C-terminus of the chimeric fiber via a conformationally flexible and hydrophilic glycine-serine 3 × (GGGGS) linker (Fig. 1C). The absence of aggregation for all macroscopically non-aggregated rAds was confirmed using photon correlation spectroscopy, which measures the mean hydrodynamic sizes (Z-averages, d.nm) and polydispersity indices (PDI) of nanoparticles in suspension (Fig. 1D). For non-aggregated wild type human Ad2/Ad5, the reported mean hydrodynamic size and PDI were ≈ 120 nm^22^ and ≤ 0.2^23^, respectively. Finally, consistently with previous report^19^, we confirmed no aggregation of 2 × iodixanol-purified rAds with the RGD-4C peptide inserted in the HI loop of the fiber knob domain (four independent viral preparations) (Supplementary Figure 1). The A260/A280 ratios for both iodixanol-purified and CsCl-purified rAds were in a range of 1.35–1.41, consistently with the values expected for pure Ad preparations^19,24^. The viral particle (vp) to infectious unit (IFU) ratios for CsCl-purified non-aggregated rAds (a quarter from ≈ 20 preparations) and iodixanol-purified rAds with the RGD-4C peptide inserted in the HI loop of the fiber knob domain was also comparable and less than 1:40. Altogether, visible macroscopic or invisible microscopic aggregation during 2 × CsCl gradient ultracentrifugation was detected exclusively for rAds containing the RGD-4C peptide in the HI loop of the fiber knob domain, which could be prevented by purification in 2 × iodixanol gradient.

### The cysteine residues with free thiol groups within the RGD-4C peptide in the HI loop of the fiber knob domain may be responsible for adenovirus aggregation during 2 × CsCl gradient ultracentrifugation

Interestingly, from about 20 preparations of Ad5-delta-24-RGD-4C-based rAds purified by 2 × CsCl gradient ultracentrifugation, a quarter was both in a macro- and microscopically non-aggregated state based on the visible observations and PDI measurements. In an attempt to find the reason of aggregation of rAds containing the RGD-4C peptide in the HI loop of the fiber knob domain, we first focused on modifications to a protocol for rAd amplification and viral particle release. Adenovirus amplification is accompanied by the increased metabolic activity of the infected cells and acidification of the medium at a higher rate. However, control of the pH balance of the medium did not overcome aggregation problem (not shown). Virus release into the serum-free medium versus serum-containing medium by three cycles of freeze-thawing also did not reveal any relevant differences during 2 × CsCl gradient ultracentrifugation (not shown). We also tried an alternative method of viral particle release from the infected cells by lysing cells with sodium deoxycholate and then incubating the cell lysate with benzonase degrading all forms of DNA and RNA (see “Materials and methods” section for details). However, again, macroscopic aggregation was not prevented during 2 × CsCl gradient ultracentrifugation (not shown).

**Figure 2:**
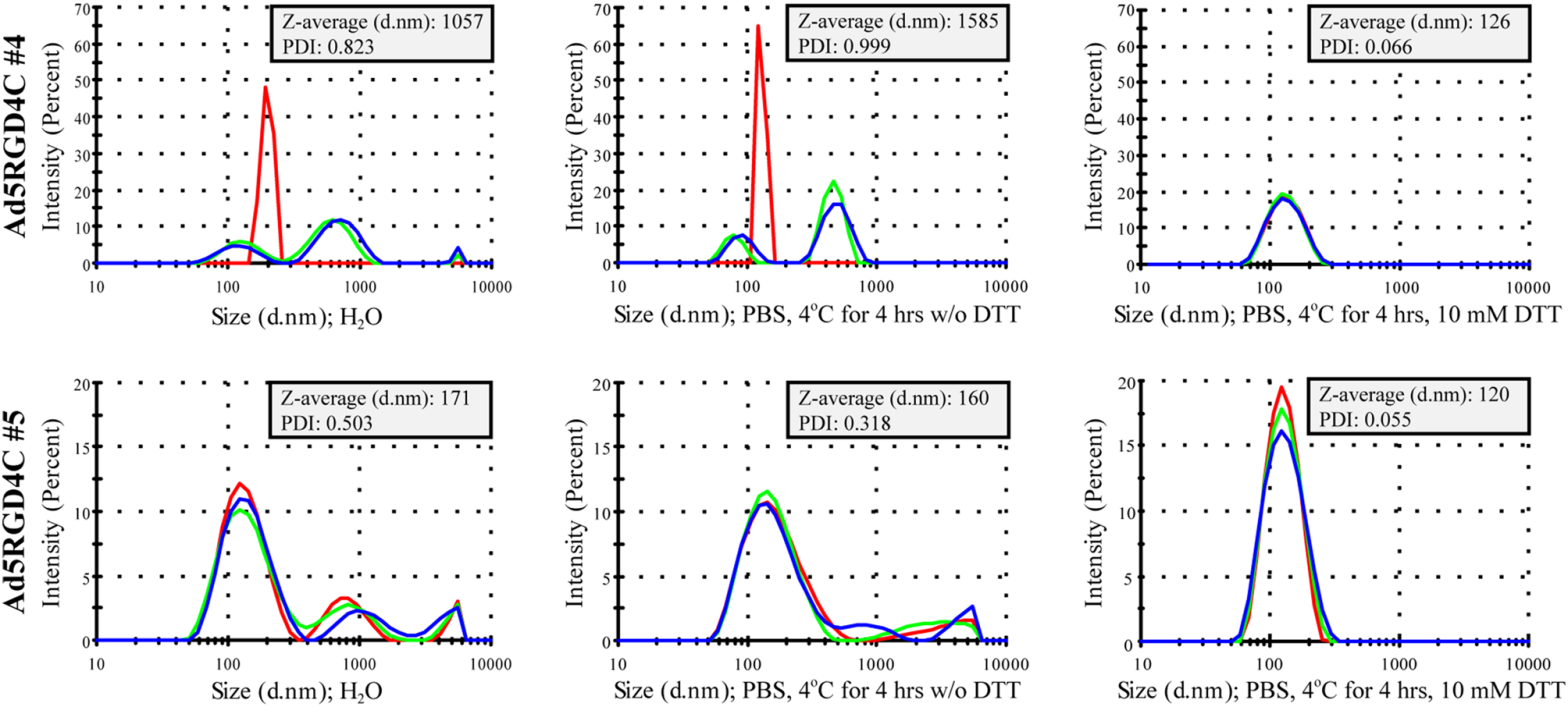
Visible macroscopic and invisible microscopic aggregates of recombinant adenoviruses with the RGD4C peptide inserted in the HI loop of the fiber knob domain could be resolved by the reducing reagent dithiothreitol. Representative macroscopically aggregated rAds (Ad5RGD4C#4 and Ad5RGD4C#5) incubated with the reducing reagent DTT showed size distributions by intensity, the intensity weighted mean hydrodynamic sizes (Z-averages, d.nm) and polydispersity indices (PDI) comparable to the values determined for non-aggregated adenoviral preparations. The measurements were carried out at a rAd concentration of 3 × 10^10^ vp in 1 ml total volume in deionized water immediately after viral aliquot sample thawing or in PBS after incubating at 4 °C for 4 h with or without the addition of 10 mM DTT. Each color curve represents an instrumental individual measurement record.

Then, we looked at the amino acid sequence of the genetically inserted RGD-4C peptide. It contains four cysteine residues at positions 2, 4, 8, and 10 (ACDC**RGD**CFCG). It was demonstrated by mass spectroscopy that in the context of the HI loop of the fiber knob domain, only cysteine residues before the RGD motif within the RGD-4C peptide formed an intramolecular disulfide bond, while thiol groups of the cysteine residues after the RGD motif were free^25^. Since the HI loops of the trimeric fibers (12 trimeric fibers per viral particle, in total 36 monomers) are highly solvent exposed, we hypothesized that aggregation could occur due to formation of the interparticle disulfide bonds under atmospheric oxygen during 2 × CsCl gradient ultracentrifugation. When we added the reducing reagent dithiothreitol (DTT, 10 mM) to the representative macroscopically aggregated rAds and incubated for 4 h at 4 °C, PDIs were reduced to the values determined for wild type Ad5 or non-aggregated rAd preparations (Fig. 2). The same results were obtained by incubating macroscopically aggregated rAds with 10 mM DTT for 1 h at 37 °C (not shown). Thus, these data imply that rAds containing the cysteine residues with free thiol groups within the RGD-4C peptide in the HI loop of the fiber knob domain aggregate due to formation of the interparticle disulfide bonds.

### A cysteine-to-glycine substitution at positions after the RGD motif within the RGD-4C peptide in the HI loop of the fiber knob domain prevented viral aggregation during 2 × CsCl gradient ultracentrifugation

**Figure 3:**
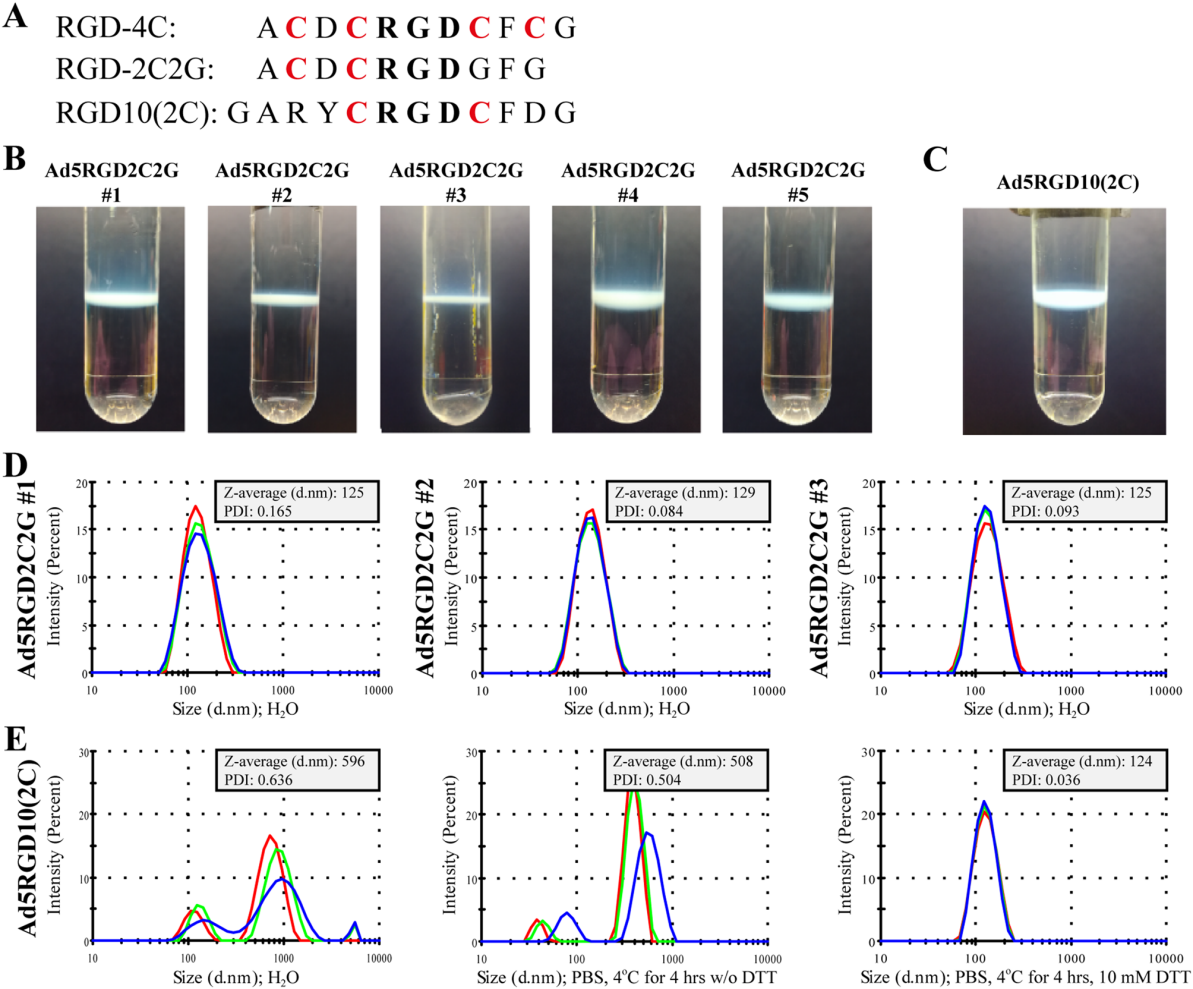
A cysteine-to-glycine substitution at positions after the RGD motif within the RGD-4C peptide in the HI loop of the fiber knob domain prevented viral aggregation during 2 × CsCl gradient ultracentrifugation. (**A**) The amino acid sequences of the RGD-4C, RGD-2C2G, and RGD10(2C) peptides containing 4, 2, and 2 cysteine residues, respectively, which were inserted in the HI loop of the fiber knob protein after a Thr residue at position 546. (**B**) No macroscopic aggregation of Ad5-delta-24-RGD2C2G with the RGD-2C2G peptide in the HI loop of the fiber knob domain during 2 × CsCl gradient ultracentrifugation (five independent viral preparations). (**C**) No macroscopic aggregation of Ad5-delta-24-RGD10(2C) with the RGD10(2C) peptide in the HI loop of the fiber knob domain during 2 × CsCl gradient ultracentrifugation. (**D**) The absence of aggregation (PDI ≤ 0.2) of Ad5-delta-24-RGD2C2G confirmed by photon correlation spectroscopy. Representative measurements are shown. (**E**) Microscopic aggregation (PDI ≈ 0.6) of Ad5-delta-24-RGD10(2C) could be resolved by incubating with DTT. The measurements of the intensity weighted mean hydrodynamic sizes (Z-averages, d.nm) and polydispersity indices (PDI) were carried out at a rAd concentration of 3 × 10^10^ vp in 1 ml total volume in deionized water immediately after viral aliquot sample thawing or in PBS after incubating at 4 °C for 4 h with or without the addition of 10 mM DTT. Each color curve represents an instrumental individual measurement record.

To further substantiate our supposition that the cysteine residues with free thiol groups within the RGD-4C peptide in the HI loop of the fiber knob domain may be the reason for rAd aggregation during 2 × CsCl gradient ultracentrifugation, we genetically substituted cysteines to glycines at positions after the RGD-motif (ACDC**RGD**GFG, RGD-2C2G) (Fig. 3A). No macroscopic aggregation of Ad5-delta-24-RGD2C2G with the RGD-2C2G peptide in the HI loop of the fiber knob domain was observed during 2 × CsCl gradient ultracentrifugation (five independent viral preparations) (Fig. 3B). The absence of aggregation (PDI ≤ 0.2) was confirmed for all viral preparations by photon correlation spectroscopy (Fig. 3D). These data clearly show that the cysteine residues with free thiol groups within the RGD-4C peptide in the HI loop of the fiber knob domain are responsible for formation of the interparticle disulfide bridges during 2 × CsCl gradient ultracentrifugation.

In addition, we constructed and analyzed Ad5-delta-24-RGD10(2C) containing the RGD10(2C) peptide in the HI loop of the fiber knob domain (Fig. 3A). Similar to the RGD-4C peptide, the RGD10(2C) peptide was discovered by a phage display peptide library technology^26^. It contains only two cysteine residues (GARYC**RGD**CFDG). A side-by-side comparison demonstrated that the synthetic double cystine-bridged RGD-4C peptide exhibited an IC50 value of 8.3 ± 2.1 nM for αVβ3 and 46 ± 11 nM for αVβ5, while the synthetic single cystine-bridged RGD10(2C) peptide exhibited IC50 of 10.3 ± 2.9 nM for αVβ3 and 102 ± 11 nM for αVβ5^27^. We assumed that if a rAd with the RGD10(2C) peptide could form the intraparticle (intrapeptide) disulfide bonds leaving no free thiol groups and exhibit comparable transduction efficiency in comparison to an RGD4C-modified rAd, it would solve the long-standing problem of aggregation during conventional 2 × CsCl gradient purification and post-purification handling without compromising enhanced infection potency. However, despite of the absence of macroscopically visible aggregation of Ad5-delta-24-RGD10(2C) during 2 × CsCl gradient ultracentrifugation (Fig. 3C), we detected strong microscopic aggregation (PDI ≈ 0.6), which could be eliminated by incubating Ad5-delta-24-RGD10(2C) vector with 10 mM DTT (Fig. 3E). These data imply that no intraparticle disulfide bonds within the inserted RGD10(2C) peptide were able to form in Ad5-delta-24-RGD10(2C).

### Recombinant adenoviruses with the RGD2C2G peptide in the HI loop of the fiber knob domain showed remarkably reduced cytolytic efficacy in comparison to the RGD4C peptide in CAR-low/negative cells

**Figure 4:**
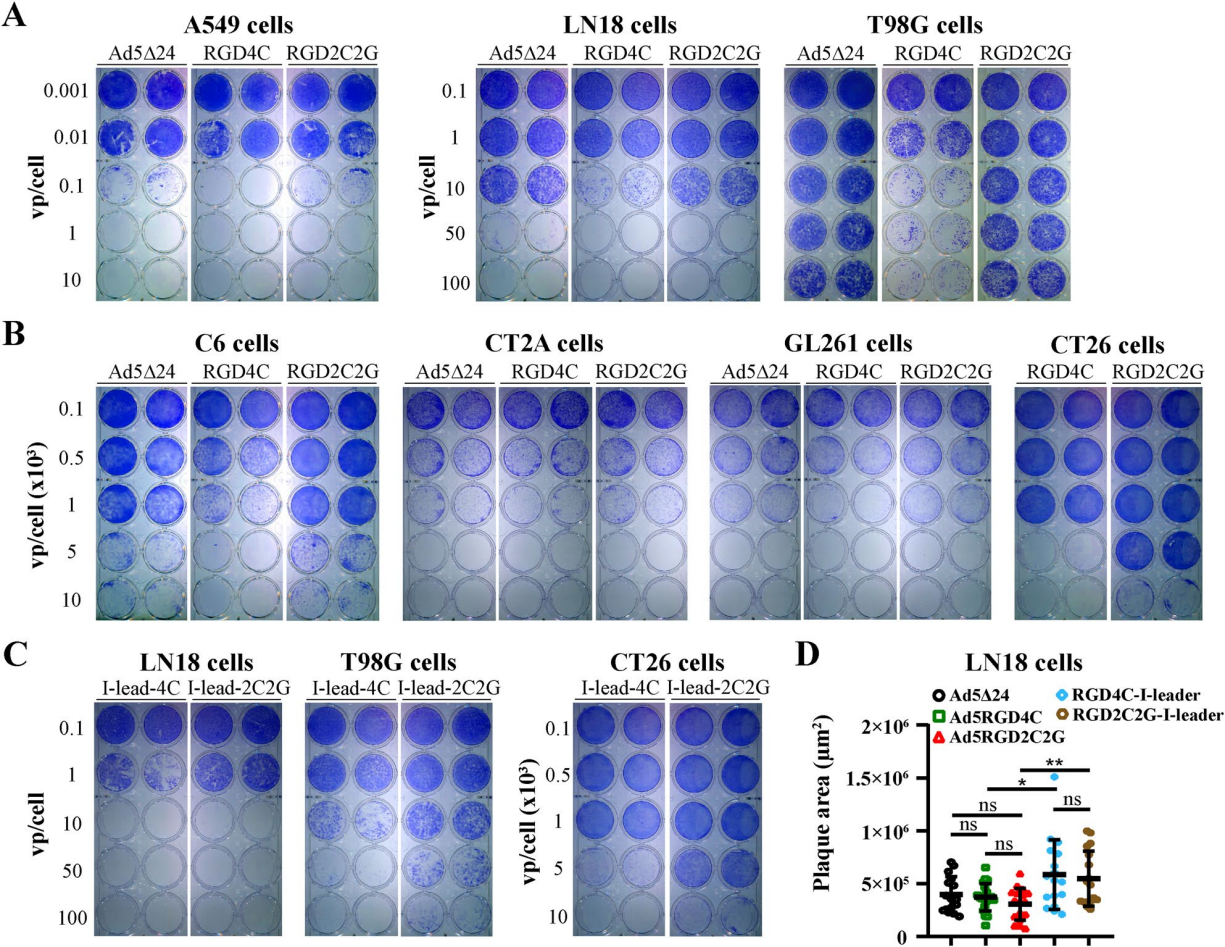
A cysteine-to-glycine substitution after the RGD motif within the RGD-4C peptide (RGD-2C2G) in the HI loop of the fiber knob domain reduced the cytotoxic efficacy in human and rodent cell lines. (**A**) Crystal violet staining of rAd infected human lung adenocarcinoma A549 and glioma LN18 and T98G cells. Cells (2.5 × 10^4^ per well, 24-well plate) were infected in suspension with rAds in a serial dilution. Eight days after infection, cells were stained with crystal violet to assess comparative cytotoxic effect of Ad5-delta-24, Ad5-delta-24-RGD4C, and Ad5-delta-24-RGD2C2G. Representative pictures of two independent experiments in duplicate with similar results are shown. (**B**) Crystal violet staining of rAd infected rat glioma C6 cells, murine glioma CT2A and GL261 cells, and murine colon carcinoma CT26 cells. Cells (2.5 × 10^4^ per well, 24-well plate) were infected in suspension with rAds in a serial dilution. Five days after infection, cells were stained with crystal violet to assess comparative cytotoxic effect of Ad5-delta-24-RGD4C and Ad5-delta-24-RGD2C2G. Representative pictures of two independent experiments in duplicate with similar results are shown. (**C**) Comparative cytotoxic effect of Ad5-delta-24-RGD4C-I-leader (Q125Stop) and Ad5-delta-24-RGD2C2G-I-leader (Q125Stop) in human and murine cells. Cells were treated and analyzed as in (**A**,**B**). (**D**) Comparison of the plaque areas of the indicated rAds in LN18 cells at day 10 post-infection (1% agarose overlay). Data are presented as mean ± SD, **p < 0.01, *p < 0.05 by unpaired two-tailed t-test with Welch’s correction. The sample sizes are indicated in the figure (n = 16–21).

**Figure 5:**
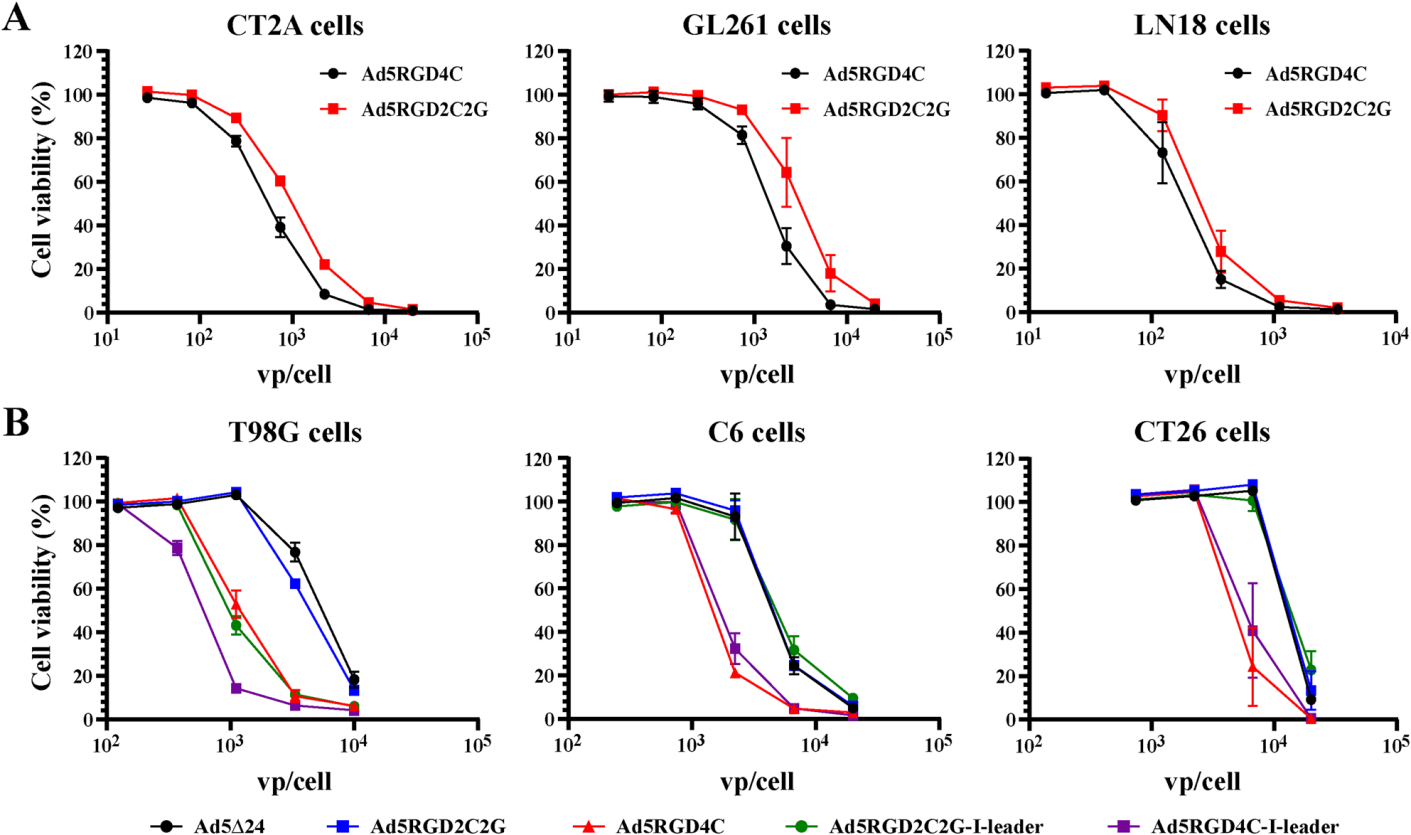
A cysteine-to-glycine substitution after the RGD motif within the RGD-4C peptide (RGD-2C2G) in the HI loop of the fiber knob domain reduced the cytotoxic efficacy in human and rodent cell lines. (**A**) The AlamarBlue cell viability assay of rAd infected glioma cells. Cells (5 × 10^3^ per well for human and 2.5 × 10^3^ per well for murine cells) were infected in suspension with rAds in a serial one-third dilution starting from 2 × 10^4^ vp/cell for murine cells and 10^4^ vp/cell for human cells. Five days after infection, the cell viability was measured. Data are presented as mean ± SD of two independent experiments in triplicate. (**B**) Comparative cytotoxic effect of Ad5-delta-24, Ad5-delta-24-RGD4C, Ad5-delta-24-RGD2C2G, Ad5-delta-24-RGD4C-I-leader (Q125Stop), and Ad5-delta-24-RGD2C2G-I-leader (Q125Stop) in human and murine cells. Cells were treated and analyzed as in (**A**). For T98G cells, cell viability was measured seven days post-infection. Data are represented as mean ± SD of two independent experiments in triplicate.

Previously, no significant difference in the transduction efficacy of Ad5RGD4C and Ad5RGD2C2G was reported in the coxsackie and adenovirus receptor (CAR)-negative MDA-MB-435S cell line, in which Ad5RGD4C demonstrated the highest transduction efficacy in comparison to wild type Ad5 among six tested cell lines^25^. If it is the case, then Ad5RGD2C2G-based rAds could be used for developing therapeutic oncolytic vectors, which would preserve increased transduction potential in a CAR-independent manner without signs of aggregation during conventional 2 × CsCl gradient ultracentrifugation and post-purification handling. To test this attractive possibility, we compared the oncolytic efficacy of Ad5-delta-24-RGD2C2G and non-aggregated Ad5-delta-24-RGD4C in human and rodent cell lines (Fig. 4). In the qualitative crystal violet staining assay, Ad5-delta-24-RGD4C was significantly more cytotoxic in human T98G glioma cells, rat C6 glioma, and murine CT26 colon carcinoma cells, while less significant differences were observed in human lung adenocarcinoma A549 and glioma LN18 cells, and in murine CT-2A and GL261 glioma cells (Fig. 4A,B). In human cells, Ad5-delta-24-RGD2C2G and Ad5-delta-24 with the wild type fiber demonstrated comparable cytopathic effects (Fig. 4A). For a side-by-side comparison of rAds, we used the physical titers (vp/cell). However, the vp/IFU ratios for the tested viral preparations were different (24:1 for Ad5-delta-24-RGD4C versus 89:1 for Ad5-delta-24-RGD2C2G versus 4.5:1 for Ad5-delta-24) (Supplementary Table 2). To exclude possible skewing of the results, we also compared Ad5-delta-24-RGD2C2G-I-leader (Q125Ter) and non-aggregated Ad5-delta-24-RGD4C-I-leader (Q125Ter), which preparations had the similar vp/IFU ratios (23:1 versus 26:1, Supplementary Table 2). These rAds additionally contain a mutation changing a Gln codon at amino acid 125 to a stop codon, resulting in the truncation of 21 amino acids from the C-terminus of the i-leader protein^28^. This and similar truncation mutations in the *i-leader* sequence did not affect virus yield or production kinetics but increased kinetics of virus release/spread from human tumor cells, including glioma cells^21^. Consistently, we observed reduced cytotoxic efficacy of Ad5-delta-24-RGD2C2G-I-leader (Q125Ter) in LN18, T98G, and CT26 cells (Fig. 4C). Additionally, in the plaque assay, we compared the spread efficiency of all five rAds in A549 and LN18 cells (Fig. 4D and Supplementary Figure 2). However, no differences in the mean plaque areas between Ad5-delta-24, Ad5-delta-24-RGD4C, and Ad5-delta-24-RGD2C2G as well as between Ad5-delta-24-RGD4C-I-leader (Q125Ter) and Ad5-delta-24-RGD2C2G-I-leader (Q125Ter) were observed (Fig. 4D and Supplementary Figure 2). Unfortunately, T98G cells were found to be not appropriate for the plaque assay, whereas the tested rodent cell lines do not support adenovirus reproduction and/or spread (not shown). Finally, reduced cytotoxic efficiency of the RGD2C2G-modified rAds in T98G, C6, and CT26 cells but not LN18 cells was confirmed in the quantitative alamarBlue cytotoxicity assay (Fig. 5A,B and Supplementary Table 3). Moreover, we also confirmed differences in the cytotoxic efficacy between Ad5-delta-24-RGD4C and Ad5-delta-24-RGD2C2G in CT-2A (IC50, 555.1 vp/cell versus 991.3 vp/cell) and GL261 (IC50, 1480 vp/cell versus 3085 vp/cell**)** murine glioma cells (Fig. 5A). Taken together, the RGD4C-modified rAds were more efficient in the majority of tested cell lines in comparison to the RGD2C2G-modified rAds.

To provide a clue on the differences in the transduction efficacy and, as a result, cytotoxicity between the RGD4C- and RGD2C2G-modified rAds in human T98G cells on the one hand and A549 and LN18 cells on the other hand, we analyzed the cell surface expression levels of CAR and integrins αVβ3 and αVβ5 by flow cytometry (Supplementary Figure 3). In contrast to A549 and LN18 cell lines (≈ 90% and ≈ 50% of CAR-positive cells, respectively), CAR-positive T98G cells were represented by a small fraction (≈ 8%), while all the cell lines abundantly expressed αVβ5 (> 80–90%) and αVβ3 (> 80–90%, except A549: ≈ 5%) integrins. Since insertion of the RGD-4C peptide in the HI loop of the fiber knob domain enhances transduction of CAR-negative cells^1^, the variability in cytotoxicity between the RGD4C- and RGD2C2G-modified rAds in the tested human tumor cells may be attributed to the expression levels of CAR.

### Both Ad5-delta-24-RGD4C and Ad5-delta-24-RGD2C2G were equally effective in the murine immunocompetent, syngeneic, orthotopic CT-2A glioma model

**Figure 6:**
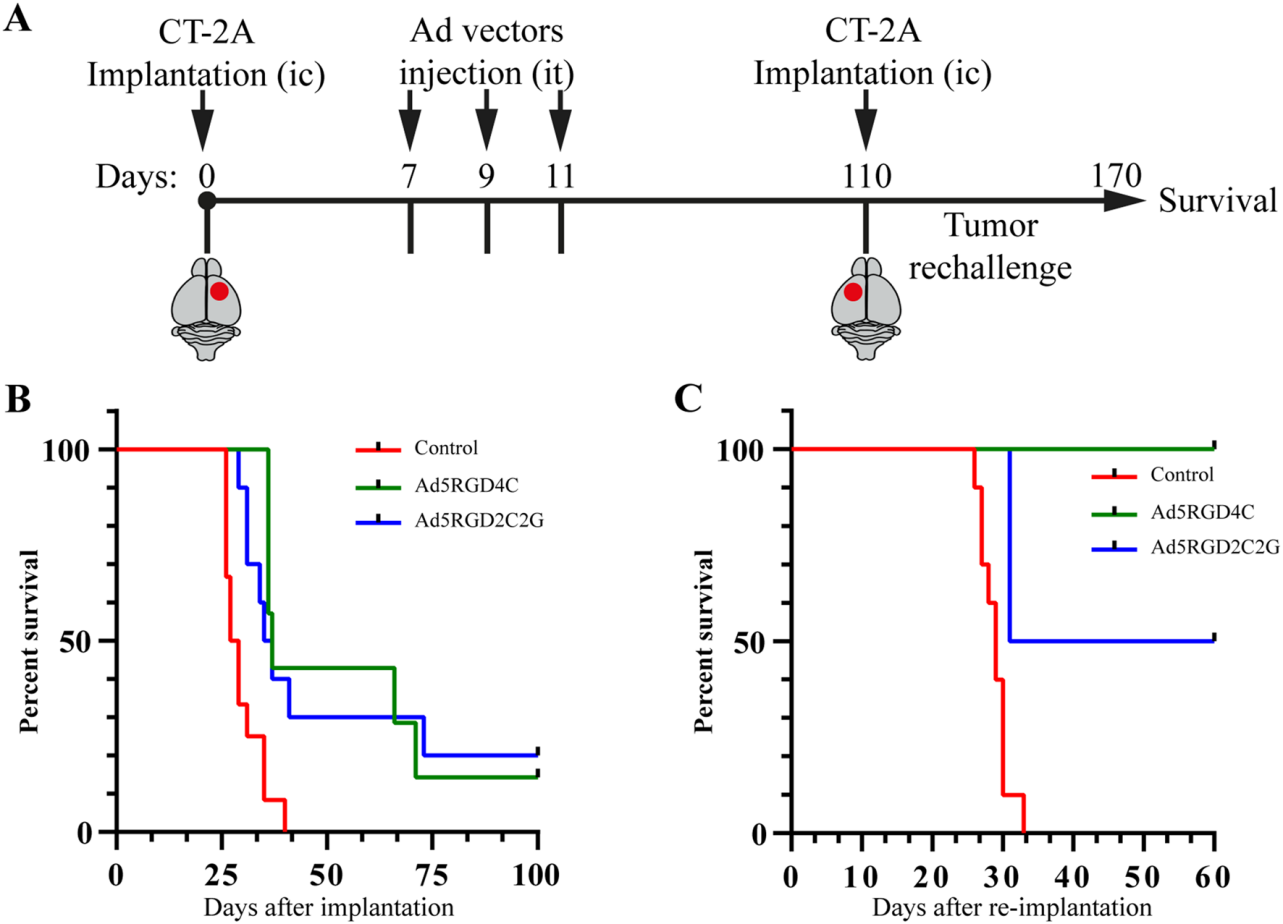
Ad5-delta-24-RGD4C and Ad5-delta-24-RGD2C2G were equally efficient in prolonging the survival of CT-2A glioma-bearing mice. (**A**) Schema of the in vivo study. CT-2A cells (5 × 10^4^ cells/5 µl) were implanted intracranially (ic) in the right striatum of syngeneic immunocompetent C57BL/6 mice using a stereotaxic system. Recombinant adenoviruses (1 × 10^10^ vp; 5 µl) were intratumorally (it) injected on day 7, 9, and 11 after tumor cell implantation (n = 7, Ad5RGD4C; n = 10, Ad5RGD2C2G). Ad buffer was used as a control (n = 12). (**B**) Survival plots of the experiment described in (**A**) showed prolonged survival with three long-term survivors (> 100 days) in the rAd-treated groups. Log-rank test: Ad5-delta-24-RGD4C vs Ad buffer, p = 0.0008; Ad5-delta-24-RGD2C2G vs Ad buffer, p = 0.0051; Ad5-delta-24-RGD4C vs Ad5-delta-24-RGD2C2G, p = 0.7598. (**C**) CT-2A rechallenge experiment with tumor cells (5 × 10^4^ cells/5 µl) implanted into the contralateral hemisphere (left striatum) of the long-term survivors (n = 1, Ad5RGD4C; n = 2, Ad5RGD2C2G). No tumor reappearance was detected in two out of three mice during a > 60-days observation period.

We compared the therapeutic efficacy of Ad5-delta-24-RGD4C and Ad5-delta-24-RGD2C2G in the immune competent, syngeneic, orthotopic murine CT-2A glioma model. Mice received intratumoral injections of viruses at doses 1 × 10^10^ vp at days 7, 9, and 11 after inoculation of glioma cells (Fig. 6A). The virus-treated mice survived significantly longer than the control buffer-treated group (log rank p = 0.0008 and p = 0.0051), with no statistical difference between the virus-treated groups (log rank p = 0.7598) (Fig. 6B). Three animals from the virus-treated groups survived more than 100 days. In a glioma rechallenge experiment, the survived animals received 5 × 10^4^ CT-2A glioma cells in the contralateral hemisphere. During a > 60-days monitoring period, two from three animals survived and none from the control tumor-naïve group (Fig. 6C). The magnetic resonance imaging confirmed that the survived animals remained tumor-free (Supplementary Figure 4). Thus, despite of the difference in cytotoxicity between Ad5-delta-24-RGD4C and Ad5-delta-24-RGD2C2G in CT-2A cells in vitro (almost two-fold difference in IC50 values), both rAds were equally effective in the immune competent CT-2A glioma model.

## Discussion

Aggregation of individual virions has been observed for viruses of different species in the environment and cell culture preparations/buffer solutions^29^. Since viral aggregation increases resistance to disinfectants^30^ and may interfere with high-quality production of vectors for gene therapy and vaccine^31^, this phenomenon has been studied for decades. Viral aggregates may vary from a few to hundreds of virions. The electrostatic and hydrophobic forces play a major role in aggregate formation. Neutralization of a net virus surface charge (i.e., repulsive electrostatic forces) by adjusting pH near or below the viral isoelectric point resulted in irreversible aggregation^32^. For instance, for human Ad2, Ad4, and Ad5, the isoelectric point pH values were determined as 3.5–4.0, 2.6, and 4.5, respectively^22^. In addition to pH, changes in temperature (i.e., Brownian movement), virus concentration, salt concentration, type and concentration of cations and anions (ionic strength of the solution), organic matter, and polyelectrolytes all influence viral aggregation-disaggregation process^32^. Interestingly, it was reported that an aggregated state (evaluated by the absorbance ratios of 320/260 and 340/260 nm) of purified rAd5-based vectors had no influence on the transduction efficacy and cytotoxicity in vitro and in rats after i.v. administration^33^. Adenovirus aggregation is generally a reversible process (except at pH near or lower than isoelectric point). However, it is not the case with the clinically advanced Ad5-delta-24-RGD, aggregating irreversibly due to a specific modification in the viral capsid.

The rAds with the RGD-4C peptide in the HI loop of the fiber knob domain extensively aggregated during 2 × CsCl gradient ultracentrifugation, while aggregation was not observed in the same conditions for non-modified rAds, fiber-chimeric Ad5/35 or rAd with the fiber modified with 21 polylysines^19^. Consistently with this pivotal report, we detected neither macroscopic nor microscopic aggregation (polydispersity index measurements) for fiber-unmodified rAds (two preparations) and fiber-chimeric Ad5/3-delta-24-based (four preparations) and Ad5/35-delta-24-based viruses (six preparations), while Ad5-delta-24-RGD-based viruses frequently exhibited irreversible aggregation (three quarters from about twenty preparations), which could be prevented using iodixanol gradient ultracentrifugation (four preparations).

Developing an adenovector-targeting platform based on a combination of genetically introduced cysteines at solvent-exposed positions of the capsid and chemical viral particle modifications by controlled covalent coupling to thiol groups of protein and nonprotein ligands, Kreppel et al. observed that during purification by 2 × CsCl gradient ultracentrifugation, rAd with the short cysteine-containing LICCCCCID peptide (five cysteine residues) inserted in the HI loop of the fiber knob domain formed macroscopically visible aggregates, which were not observed in the case of the LIGGGCGGGID peptide (three cysteine residues) and LIGCGCGCGID peptide (one cysteine residue) inserted in the same position^23^. However, analysis of viral preparations by photon correlation spectroscopy determining particle sizes and particle size distribution in a suspension revealed that these cysteine-containing rAds but not an unmodified control vector formed invisible strong microscopic aggregates, which could be quantitatively resolved by adding the reducing reagents, dithiothreitol (DTT, 10 mM) or tris(2-carboxyethyl)phosphine (TCEP, 10 mM). Moreover, aggregation could be prevented during 2 × CsCl gradient ultracentrifugation by adding DTT directly to a CsCl gradient. Thus, the genetically introduced thiol groups in the HI loop of the fiber knob domain were highly reactive and prone to form the interparticle disulfide bonds^23^.

The RGD-4C peptide (ACDC**RGD**CFCG) contains four cysteine residues. Previously, Majhen et al. were specifically interested in how many intramolecular disulfide bonds and in which configuration were formed within the RGD4C peptide inserted in the HI loop of the fiber knob domain, and whether the disulfide bond(s) in the RGD4C peptide influenced the retargeting potential of RGD4C-modified rAd5^25^. The mass spectroscopy analysis revealed that in the context of the HI loop of the fiber knob domain, only cysteine residues before the RGD motif within the RGD-4C peptide formed an intramolecular disulfide bond. However, the authors have not dealt with the aggregation issue^25^. In the present study, we proved that the cysteine residues with free thiol groups after the RGD motif within the RGD-4C peptide inserted in the HI loop of the fiber knob domain were responsible for rAd aggregation during 2 × CsCl gradient ultracentrifugation. First, we were able to reverse macroscopic and microscopic aggregation by adding the reducing agent DTT (10 mM) to the aggregated rAds. Second, a genetic substitution of the cysteine residues with free thiol groups to glycines within the RGD-4C peptide in the HI loop of the fiber knob domain (RGD-2C2G, ACDC**RGD**GFG) prevented aggregation (five independent vector preparations). Currently, it is not known whether an intramolecular disulfide bond between the cysteine residues before the RGD motif within the RGD-4C peptide in the HI loop of the fiber knob domain is formed intracellularly despite the reducing cyto- and nucleoplasmic milieu where Ad virions assemble or outside the cell upon virus egress into the oxidizing extracellular medium^25^. Now, it is obvious that the cysteine residues with free thiol groups after the RGD motif within the RGD-4C peptide in the HI loop become non-enzymatically oxidized by atmospheric oxygen resulting in formation of the interparticle disulfide bonds and irreversible rAd aggregation during 2 × CsCl gradient ultracentrifugation. However, rAds with the RGD-4C peptide in the HI loop of the fiber knob domain do not aggregate during iodixanol gradient ultracentrifugation (^19^ and this study). We did not reveal any reducing properties of iodixanol by incubating an aggregated RGD4C-modified rAd with 25% iodixanol solution at 4 °C or 37 °C followed by PDI measurements (not shown). We speculate that the antioxidant activity of iodixanol rather might be the reason for the lack of aggregation. Iodixanol was reported to exhibit antioxidant properties in in vitro free radical generating reactions and in the assays evaluating antioxidant activity of non-enzymatic antioxidants^34,35^.

Interestingly, fiber-chimeric Ad5/3-delta-24-RGD4C (two preparations) and Ad5/35-delta-24-RGD4C (one preparation) with the RGD-4C peptide fused to the C-terminus of the chimeric fiber via a glycine-serine 3 × (G4S) linker did not aggregate during 2 × CsCl gradient ultracentrifugation. Currently, we do not know how many cysteine residues with free thiol groups are present in the C-terminally located RGD-4C peptide. If to assume that at least two cysteine residues could not be engaged in formation of an intramolecular disulfide bond, then the reasonable question why these rAds did not aggregate during 2 × CsCl gradient ultracentrifugation. We suppose that formation of the interparticle disulfide bonds might be sterically and energetically unfavorable for these rAds. The flexible HI loop is exposed on the exterior of the fiber knob, while the C-terminus is located at the base of the knob, facing back towards the viral capsid^36^. Although the flexible 3 × (G4S) linker is supposed to bring the RGD-4C peptide to a more available position for interaction with the host cell surface, the RGD-4C peptide might still not be in enough proximity to allow formation of the interparticle disulfide bonds. To the point, there are controversies in the literature regarding improved transduction efficiency of Ad5/3RGD-based rAds over parental Ad5/3^37–39^. We found that depending on a cell line, Ad5/3RGD showed comparable or inferior cytolytic efficacy compared to parental Ad5/3 in human and rodent cell lines (Stepanenko et al., submitted).

In the crystal violet and alamarBlue cell viability assays, we revealed that the replication-competent RGD4C-modified rAds were more efficient in comparison to the RGD2C2G-modified rAds in human T98G glioma cells (≈ 8% of CAR-positive cells by flow cytometry), in CAR-negative rat C6 glioma and murine CT26 colorectal carcinoma cells (no expression of CAR detected by Western blot analysis^40,41^), and, to a lesser degree, in murine CT-2A and GL261 glioma cells with unknown CAR expression levels but not in CAR-positive human lung adenocarcinoma A549 (≈ 90% by flow cytometry) and glioma LN18 (≈ 50% by flow cytometry) cells. Since the RGD-4C peptide inserted in the HI loop of the fiber knob domain enhances CAR-independent transduction^1^, our data imply that a cysteine-to-glycine substitution made the RGD2C2G peptide non-functional or, at least, significantly reduced the transduction efficiency. Our results collaborate with previous observation that the nature of amino acid residues after the RGD motif in the αVβ3/αVβ5 integrin-binding peptides/proteins may be important for interaction with integrins. It was documented that many peptides and proteins with high affinity to αVβ3/αVβ5 integrins contain a negatively charged residue (Asp, Glu) or Gln, or Cys at position + 3 from the RGD motif^26^. This position is occupied by a cysteine residue in the RGD-4C-peptide and by a glycine residue in the RGD-2C2G peptide. Nevertheless, the fact that the RGD2C2G-modified rAds showed decreased efficacy compared to the RGD4C-modified rAds in CAR-low/negative cells was unexpected for us, since Majhen et al. previously reported no significant difference in the transduction efficacy of the replication-deficient Ad5RGD4C and Ad5RGD2C2G vectors expressing the β-galactosidase reporter gene in CAR-negative MDA-MB-435S cell line and concluded that the cysteine residues after the RGD motif were not crucial for retargeting to αV-integrins^25^. However, to prove this, the authors used the only cell line, MDA-MB-435S. In our study, we used a panel of cell lines, two independent methods of analysis and several independent viral preparations and recombinant viruses. The inconsistent observations between studies hardly can be explained by differences in the assays or in the replication competencies between used viruses. Based on our analysis, we conclude that the cysteine residues, or more generally, the nature of amino acid residues after the RGD motif within the RGD-4C peptide in the HI loop of the fiber knob domain does play an important role in the RGD4C peptide-integrin interactions.

Previously, it was shown that Ad5-delta-24-RGD-treated athymic mice survived significantly longer than Ad5-delta-24-treated mice with U87 intracranial xenografts^1^. Since the cytopathic efficacy of the RGD2C2G-modified rAds used in this study was reduced in CAR-low/negative cells to the levels observed for the fiber non-modified control Ad5-delta-24 virus, we believe that the RGD4C-modified rAds may be also more therapeutically potent than the RGD2C2G-modified rAds in glioma orthotopic xenografts in immune deficient mice, especially in CAR-low/negative xenografts. On the other hand, there is a growing number of studies on different oncolytic viruses, which reported no correlation between viral permissivity, replication capacity, spread, and/or cytopathic effects in vitro and the antitumor effects and survival in the immune competent murine models^42^. For instance, several genetically modified oncolytic herpes simplex virus type-1 viruses showed different replication capacities and cytopathic effects in vitro^43^. However, in an immune competent murine model of breast cancer, tumor regression and survival of mice were similar with all tested viruses, indicating that in vitro cytolytic properties may be poor prognostic indicators of in vivo antitumor activity in an immune competent murine model^43^. The similar observations were reported for vaccinia virus Ankara^44^, reovirus^45^, vesicular stomatitis virus^46^, and newcastle disease virus^47^ in different immune competent murine models. We compared the therapeutic efficacy of Ad5-delta-24-RGD4C and Ad5-delta-24-RGD2C2G in the immune competent, syngeneic, orthotopic murine CT-2A glioma model and found that both viruses were equally effective. Previously, it was reported that the therapeutic effect of Ad5-delta-24-RGD4C depended primarily on the antitumor immune responses in another immune competent, syngeneic, orthotopic murine GL261 glioma model^16,48^. Consistently with previous reports, we speculate that differences in the efficacy between Ad5-delta-24-RGD4C and Ad5-delta-24-RGD2C2G in vitro were not replicated in vivo due to a primary role of antitumor immune responses in the therapeutic efficacy of oncolytic viruses in the immune competent murine models^42–46^.

Many efforts were applied to find such buffer formulations that would prevent aggregation and increase purity of rAds during purification, and improve stability during long-term storage. For instance, in a chromatography-based purification process, co-purification of the host cell DNA with rAd particles from the benzonase-treated clarified lysate was found to be due to association with Ad5 aggregates. Ad5 aggregation could be prevented by using the nonionic detergent polysorbate-80 throughout the purification process, whereas high spiked NaCl concentrations dissociated host cell DNA/Ad5 complexes resulting in clearance of residual DNA below a detectable limit (5 pg/10^11^ viral particles)^49^. Another example is a liquid adenovirus formulation for use in gene therapy and vaccine applications allowing long-term storage (at least 2 years) at 4 °C and compatible with parenteral administration. It comprises a buffer (Tris–HCl), a sugar (sucrose), a salt (NaCl), a divalent cation (MgCl_2_), a non-ionic detergent (polysorbate-80), a free radical scavenger (ethanol and histidine) and/or chelating agent (ethylenediaminetetraacetic acid, EDTA) to inhibit free radical oxidation^50^. For rAds with the RGD-4C peptide in the HI loop of the fiber knob domain, an efficient iodixanol-based purification protocol preventing irreversible virus aggregation has also been established^19^. In the present study, we finally provided a mechanistic explanation on the long-standing issue how and why rAds with the RGD-4C peptide in the HI loop of the fiber knob aggregate during CsCl but not iodixanol gradient ultracentrifugation. This new insight may have implications in large-scale purification of the RGD4C-modified rAds for clinical trials. It was shown that premature oxidation of thiols and aggregation of rAds with the genetically introduced cysteine residues at the solvent-exposed surface of rAd particle during 2 × CsCl purification may be prevented in an inert argon atmosphere in the absence of reducing reagents^23,51^. Hence, saturating the buffers with argon gas may be an option during large-scale purification and post-purification storage of the RGD4C-modified rAds.

## Materials and methods

### Cell lines

Human embryonic kidney HEK293, lung adenocarcinoma A549, glioma T98G and LN18, rat glioma C6 (ATCC), murine glioma CT-2A and GL261 (kindly provided by Dr. Zsolt Ruzsics, Institute of Virology, University of Freiburg, Germany) were grown in DMEM (Gibco) containing 10% FBS (Hyclone). Murine CT26 colon carcinoma cells (ATCC) were cultured in RPMI-1640 (Gibco) supplemented with glucose (4.5 g/l), l-glutamine (2 mM), sodium pyruvate (1 mM), HEPES (10 mM), and 10% FBS. All cell growth media were supplemented with 100 U/ml penicillin and 100 μg/ml streptomycin. All the cell lines were maintained in an incubator at 37 °C in a humidified atmosphere of 95% air and 5% CO_2_ and tested for mycoplasma contamination. All the human cancer cell lines were authenticated by short tandem repeat (STR) analysis in 2019.

### Construction of recombinant adenoviruses

We first assembled a cloning vector, pSC101-CmR-PacI, containing the pSC101 low copy origin of replication with the partition (par) locus (derived from pSC101-Timer plasmid, Addgene #103057) and chloramphenicol resistance gene, and two PacI sites for releasing the adenoviral genome. The full-length genome of human adenovirus 5 (strain Adenoid 75, VR-5, ATCC) was incorporated into this cloning vector as described in^52^ using RecET-mediated linear–linear homologous recombination in *E. coli* GB05-dir strain (Gene Bridges, Heidelberg, Germany). To modify the adenoviral genome, a selection-counterselection *rpsL*-neo cassette (from the “Counter-Selection BAC Modification Kit”, Gene Bridges) and λ-Red-mediated linear-circular homologous recombination in *E. coli* GB08-red strain (Gene Bridges) were exploited. For recombination, 30 µl of overnight culture were inoculated into 1.4 ml low salt LB broth (5 g/l NaCl) containing streptomycin (linear–linear homologous recombination) or chloramphenicol (linear–circular homologous recombination to maintain a plasmid) and grown in 1.5 ml tubes with needle-punctured caps at 950 rpm, 37 °C in a thermoshaker TS-100C (BioSan, Latvia). At an absorbance of ≈ 0.4, 50 µl of 10% l-arabinose was added (final 0.35% w/v) to induce *RecET* or *redγβα/recA* expression in *E. coli* GB05-dir and *E. coli* GB08-red strains, respectively. After 35 min (an absorbance ≈ 0.7–0.8), cells were harvested by centrifugation at 5900×*g* at room temperature (all procedures afterwards were carried out at room temperature irrespectively of a type of recombination), washed twice with autoclaved ddH_2_O and once with 10% (v/v) glycerol, and resuspended in a final volume of 30–35 μl in 10% glycerol. For linear–linear homologous recombination, the adenoviral genomic DNA (≈ 500 ng) and the linearized cloning vector (≈ 500 ng) with homology arms were added to the cells, mixed and transferred to an electroporation cuvette with 0.1 cm gap (Bio-Rad), and electroporation was carried out with a MicroPulser (Bio-Rad) at a constant of 1.7 kV (Ec1 program). For linear-circular homologous recombination, pSC101-CmR-Ad5 plasmid was maintained inside the cells by chloramphenicol selection and only PCR product, annealed complementary oligos or single-strand oligo (≈ 500 ng) were added for electroporation. The cells were immediately removed from a cuvette by mixing with pre-warmed 1 ml low salt LB medium, and then incubated in a thermoshaker at 37 °C, 950 rpm, for 1.5 h. The cells were collected by centrifugation at 5900×*g* and usually all plated on a low salt LB agar containing the appropriate antibiotics. Sometimes, a 1/10th–1/100th part of cells was sufficient to plate for preventing extensive colony background/formation of a bacterial lawn. Antibiotics were used at the following final concentrations in LB and solid media: chloramphenicol (15 μg/ml), kanamycin (30 μg/ml), and streptomycin (200 μg/ml). A routine diagnostic restriction digest of rAds after each round of recombination was carried out using EcoRV and XhoI (Thermo Scientific). Screening of clones was conducted using PCR followed by sequencing. All the oligonucleotides (Supplementary Table 1) were ordered from Evrogen (Moscow, Russia) or Syntol (Moscow, Russia) as unpurified (for screening colony PCR or sequencing) or HPLC/PAGE-purified (for recombination) depending on the length of oligo. To perform in-silico linear–linear and linear-circular homologous recombination, a Molecular Cloning Designer Simulator (MCDS) software was used^53^.

### DNA amplification and gel extraction

For amplification of DNA fragments for recombination, Phusion Green Hot Start II High-Fidelity PCR Master Mix (Thermo Scientific) was used according to the manufacture’s recommendations. The predicted annealing temperatures for each oligo pair were determined using an online T_m_ Calculator (Thermo Scientific). Using the QIAquick Gel Extraction Kit (Qiagen), PCR fragments were isolated from 0.8% agarose stained with SYBR Safe DNA Gel Stain (Invitrogen) and visualized with Safe Imager 2.0 Blue-Light Transilluminator (Invitrogen). Before electroporation, gel-extracted PCR products were desalted using membrane filters (#VSWP02500, Millipore). For colony PCR screening, DreamTaq Green PCR Master Mix (Thermo Scientific) or PCR ScreenMix (Evrogen) were used. Purified genomic DNA of human adenovirus 3 (strain G.B., VR-847, ATCC) and adenovirus 35 (strain Holden, VR-718, ATCC) were used as a template for constructing fiber-chimeric Ad5/3 and Ad5/35 vectors.

### Transfection, adenovirus amplification, purification, storage, and tittering

HEK293 cells were seeded onto 3 cm plates (two for each rAd rescue) and transfected at 70–90% confluency with the PacI-linearized adenoviral genome (2.5 µg per plate as measured by densitometry in gel, purified with ethanol plus glycogen precipitation) using 5 µl of reagent P3000 and 5 µl of Lipofectamine 3000 (Invitrogen). After 3–5 days, both cells and supernatant were collected, three times freeze-thawed, centrifuged at 2000×*g* for 5 min, and all supernatant was added to one near-confluent T75 flask with A549 cells. Depending on a vector and the efficiency of transfection, we usually observed signs of cytopathic effects after 3–10 days. When at least a half of the cells was rounded, both cells and supernatant were collected, three times freeze-thawed, centrifuged at 2000×*g* for 10 min, and 1/8th–1/10th part was diluted and added to five near-confluent T175 flasks. The infected cells were collected within 2–3 days. After three cycles of freezing and clearing by centrifugation at 2000×*g* for 10 min, the cell lysate solution (≈ 5 ml of a rescued virus in DMEM without serum) was transferred on the top of a CsCl step gradient (5 ml of 1.27 g/cm^3^ and 3 ml of 1.41 g/cm^3^ ρCsCl in 20 mM HEPES, 150 mM NaCl buffer, pH 7.6) in 13.2 ml Beckman ultra-clear ultracentrifuge tubes. (We also tried an alternative method of viral particle release from the infected cells before ultracentrifugation. The cell pellet was resuspended in 10 ml of 50 mM Tris–HCl, pH 8.0, 1 mM MgCl_2_, 0.4% sodium deoxycholate and agitated for 10 min. Then, 250U of benzonase nuclease (Millipore) was added to the cell lysate and incubated at 37 °C for an hour to reduce the viscosity. The supernatant was collected by centrifugation for 20 min at 1400×*g*. The ultracentrifugation tubes were filled to the top with HEPES buffer and centrifuged in a Beckman SW40Ti rotor at 28,000 rpm (100,000×*g*) in a Beckman Avanti 90L ultracentrifuge at 8 °C for an hour without the brake. Two rounds of ultracentrifugation were conducted. The virus bands were usually collected in a volume of ≈ 500 µl and dialyzed in Slide-A-Lyzer 10 K dialysis cassettes (Thermo Scientific) against 1L of storage buffer (5 mM Tris, 75 mM NaCl, 1 mM MgCl_2_, 5% sucrose (w/v), 0.005% PS-80, pH 8.0) at 4 °C for 2 h and then overnight. The viruses were aliquoted and stored at − 70 °C.

For rAd purification in 2 × iodixanol discontinuous density gradient, we referred to the available protocol^54^. For Beckman ultra-clear 13.2 ml tubes, we used the following volumes of step gradients: Sol 4 (15%, 1.5 ml), Sol 3 (25%, 3 ml), Sol 2 (40%, 3 ml), and Sol 1 (54%, 0.5 ml). Ultracentrifugation was conducted at 35,000 rpm (155,000×*g*) in a SW40 Ti rotor at 8 °C for an hour. After the second round of ultracentrifugation, iodixanol from the purified virus/iodixanol fraction was first removed by size-exclusion column chromatography using virus storage buffer and Zeba spin desalting column, 7K MWCO, 2 mL (Thermo Scientific) and then remaining trace amounts by overnight dialysis against 1 L of Ad storage buffer as described above.

The physical titer (optical particle units, OPU) was determined by measuring an absorbance at 260 nm in the range of 0.1–1.0 OD on a NP80 spectrophotometer (Implen, Germany) using at least three dilutions. DNA was released from virions in lysis buffer (100 mM Tris, 10 mM EDTA, 0.1% SDS) in a thermoshaker at 56 °C, 500 rpm, for 10 min. The optical particle units (OPU) for rAds were calculated using the following formula: OPU/mL = (absorbance at 260 nm) × (dilution factor) × (1.1 × 10^12^). For determining the titer of infectious particles, rAds were tittered on A549 cells by Adeno-X™ Rapid Titer Kit (Takara Bio) according to the manufacturer’s instructions. The virus particle (vp) to infectious unit (IFU) ratios for preparations of rAds are listed in Supplementary Table 2.

### Dynamic light scattering/photon correlation spectroscopy

The particle size distribution (polydispersity index, PDI) of rAds was measured using a Zetasizer Nano ZS (Malvern Instruments, UK). Measurements were carried out at an rAd concentration of 3 × 10^10^ vp/ml in autoclaved deionized water or PBS in 1 ml total volume in cuvettes at 25 °C with the default instrument settings and automatic analysis.

### Flow cytometry

The cells were detached by treatment with accutase (StemCell Technologies) and washed with flow cytometry buffer, consisting of PBS supplemented with 2 mM EDTA and 1% BSA. At least 5 × 10^5^ cells were stained with conjugated antibodies at concentrations recommended by the manufacturers in a total volume of 100 µl, incubating tubes in a thermoshaker at a low rotation speed at 4 °C for an hour, then washed once with buffer and resuspended in 0.5 ml of buffer. Data were acquired employing a MoFlow XDP (Beckman Coulter) and analyzed using Summit V5.2 (Beckman Coulter). At least 1 × 10^4^ events per sample were analyzed. Antibody conjugates: CAR-FITC (clone 271, 10799-R271-F, Sino Biological, China), integrin αVβ3-APC (23C6, 304416, Biolegend), integrin αVβ5-PE (P1F6, 920008, Biolegend); isotypes: IgG-FITC (11-4614-80, eBioscience), IgG1-APC (MOPC-21, 400122, Biolegend), and IgG1-PE (MOPC-21, 400114, Biolegend). Each staining experiment was independently repeated at least two times.

### Crystal violet cytotoxicity assay

The cells were seeded to a 24-well plate (25,000 cells/well, 0.4 ml of medium with 10% FBS) and infected in suspension (50–100 µl of viral inoculum) with serial dilutions of rAds (two wells per dilution, vp/cell designated in figures). After 3–4 days post-infection for human cells, 1 ml of complete growth medium was added to each well. After 5 days post-infection for murine cells or 8 days for human cells, wells were washed once with PBS, fixed in methanol for 10 min at − 20 °C, stained with crystal violet (0.05% in aqueous 20% methanol) for 30 min, washed with distilled water, dried and scanned with an Epson Perfection V370 scanner. Each experiment was repeated at least two times.

### AlamarBlue cytotoxicity assay

The cells (5000 cells/well for human cell lines and 2500 cells/well for rodent cells) were seeded to 96-well plates (80 µl of medium with 10% FBS) and infected in suspension (20 µl of viral inoculum) with serial dilutions of rAds (vp/cell designated in figures). Next day, 100 µl of fresh complete medium was added to each well. At the indicated days of analysis, 100 µl of complete medium with 10% resazurin/alamarBlue viability reagent (Invitrogen) was added to each well. A 0.15 mg/ml solution of resazurin (sodium salt, pure, certified, #418900010, Acros Organics, China) in PBS was used (stored frozen and aliquoted for single use). After 4 h of incubation, fluorescence was measured in a PerkinElmer EnSpire multimode plate reader with set excitation and emission wavelengths of 560 nm and 590 nm, respectively. Each experiment was repeated at least two times in triplicates.

### Plaque assay

A549 and LN18 monolayers seeded in 6-well plates were infected with serial dilutions of rAds. Two hours post-infection, the viral inoculum (400 µl) was removed and cells were covered with 3 ml of a mix of DMEM/5% FBS/1% agarose. Two days later, DMEM/5% FBS overlay was added. To evaluate the plaque size, monolayers were stained six (A549) or ten (LN18) days post-infection by incubating with 0.5 mg/ml thiazolyl blue tetrazolium bromide (MTT) at 37 ºC for 4 h. The plaques were photographed at 50 × with the Leica DM3000 microscope and quantified with a Leica Application Suite microscope software.

### Intracranial syngeneic murine CT-2A glioma modeling and treatment

The housing conditions and all the experimental procedures were set up and maintained in accordance with Directive 2010/63/EU of 22 September 2010 and approved by the local ethical committee of V.P. Serbsky National Medical Research Center. The study was carried out in compliance with the ARRIVE guidelines. Eight-week-old female immune competent C57BL6 mice maintained in individually ventilated cages (Tecniplast, Italy) were anesthetized by intraperitoneal injection of 50 mg/kg zoletil with 5 mg/kg xylazine, and intracranially implanted with 5 × 10^4^ CT-2A murine glioma cells in the right hemisphere using a stereotaxic instrument (Stoelting, USA) at stereotaxic coordinates of bregma, 2 mm lateral, 1 mm caudal and 3 mm ventral. 5 μl of cell suspension in DMEM was delivered at a depth of 3 mm using a 100 μl Hamilton microsyringe and a 2 pt style needle at the rate of 0.5 μl/min. The microsyringe was removed at a rate of 0.5 mm/min. Mice were randomly allocated to groups (n = 10, Ad5RGD4C; n = 10, Ad5RGD2C2G; n = 12, control/Ad buffer). The sample sizes were chosen according to standard practice in the field. At days 7, 9, and 11 after tumor cell implantation, mice were injected intratumorally with either 5 μl of adenovirus storage buffer or 1 × 10^10^ vp of rAds. Three animals from the Ad5RGD4C group were eventually excluded from the study since the animals died prematurely before receiving all three injections of rAd. Mice were monitored over time and euthanized when they demonstrated moribund behavior. After 100 days post-tumor implantation, the survived animals were again intracranially implanted with 5 × 10^4^ CT-2A murine glioma cells in the left (i.e., contralateral) hemisphere and monitored for > 60 days. After about 30 days and 100 days after tumor implantation, and 60 days after tumor rechallenge, T2-weighted brain magnetic resonance imaging (Brucker, USA) was applied to confirm the presence/absence of tumor growth.

### Statistics

The data were expressed as mean ± standard deviation (SD). An unpaired two-tailed Student’s *t* test with Welch’s correction where appropriate was used for evaluating the differences between groups in quantitative studies of cultured cells. The number of viral particles per cell required to produce 50% inhibition (IC50) was estimated from a dose–response non-linear regression curve ([Inhibitor] vs. normalized response). The survival rates in the different animal treatment groups were compared using the log-rank test. The statistical analysis was performed with GraphPad Prism v8 (GraphPad Software Inc., USA). **p < 0.01, *p < 0.05.

## Supplementary Information

Supplementary Information.
