# Supplementary material for: A new insight into aggregation of oncolytic adenovirus Ad5-delta-24-RGD during CsCl gradient ultracentrifugation
Source: Sci Rep. 2021 Aug 9;11:16088. doi: 10.1038/s41598-021-94573-y (PMC8352973; doi:10.1038/s41598-021-94573-y)
Supplement: Supplementary file 1 — Supplementary Information. [file 41598_2021_94573_MOESM1_ESM.docx]

**Supplementary Information**

**______________________________________________________________________________**

**A new insight into aggregation of oncolytic adenovirus Ad5-delta-24-RGD during CsCl gradient ultracentrifugation**

Aleksei A. Stepanenko^1,2,*^, Anastasiia O. Sosnovtseva^1^, Marat P. Valikhov^1,2^, Vladimir P. Chekhonin^1,2^

^1^Department of Fundamental and Applied Neurobiology, V.P. Serbsky National Medical Research Center of Psychiatry and Narcology, The Ministry of Health of the Russian Federation, Kropotkinsky lane 23, 119034 Moscow, Russia. [a.a.stepanenko@gmail.com](mailto:a.a.stepanenko@gmail.com)

^2^Department of Medical Nanobiotechnology, Institute of Translational Medicine, N.I. Pirogov Russian National Research Medical University, The Ministry of Health of the Russian Federation, Ostrovitianov str. 1, 117997 Moscow, Russia.


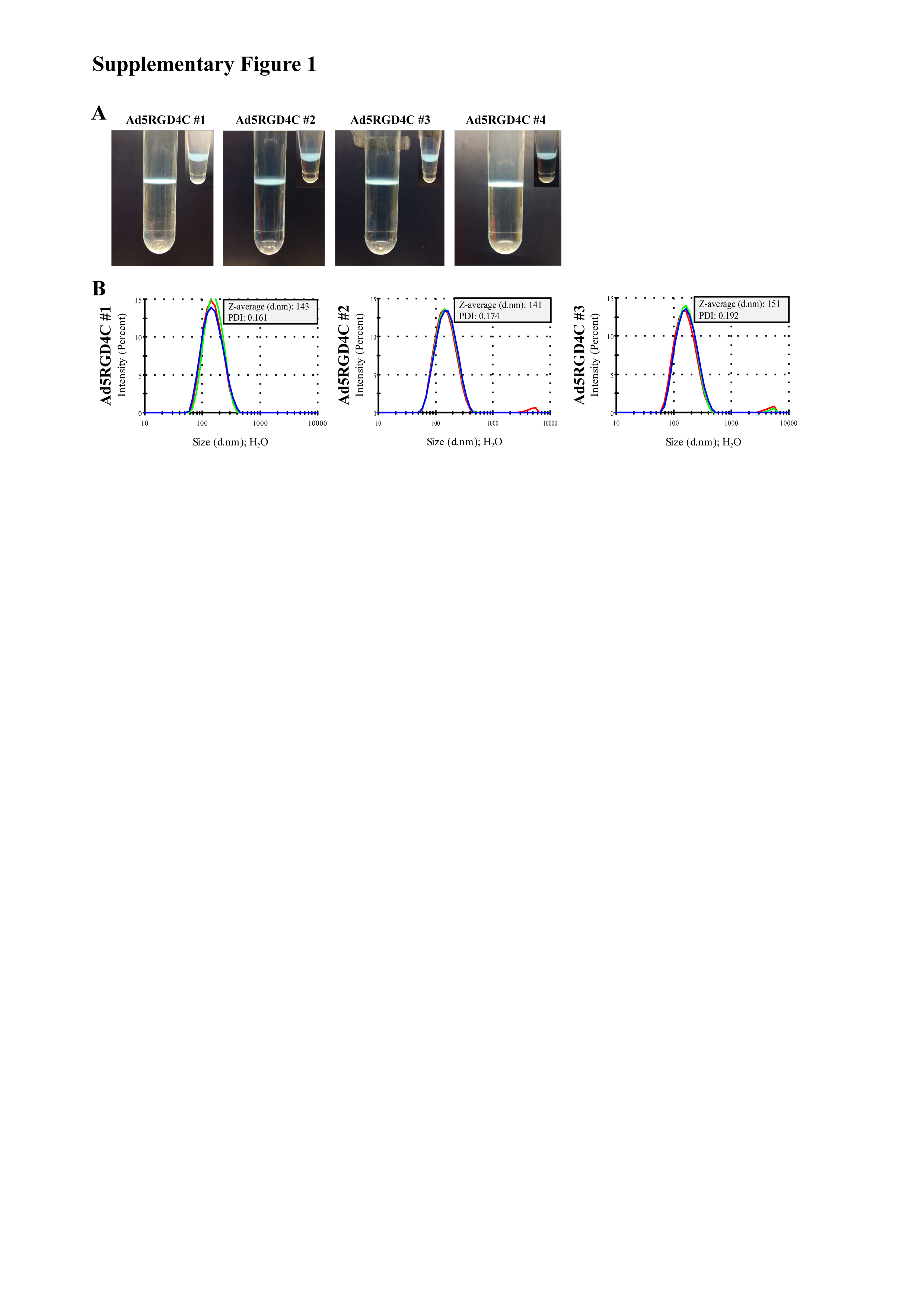
**Supplementary Figure 1.** **No aggregation of iodixanol-purified recombinant adenoviruses with the RGD-4C peptide inserted in the HI loop of the fiber knob domain.** (**A**) The pictures of rAds after the second round of ultracentrifugation in iodixanol gradient (four independent viral preparations) demonstrating the absence of macroscopic aggregation. (**B**) The absence of microscopic aggregation for iodixanol-purified rAds confirmed by photon correlation spectroscopy (PDI ≤0.2). Representative measurements are shown.


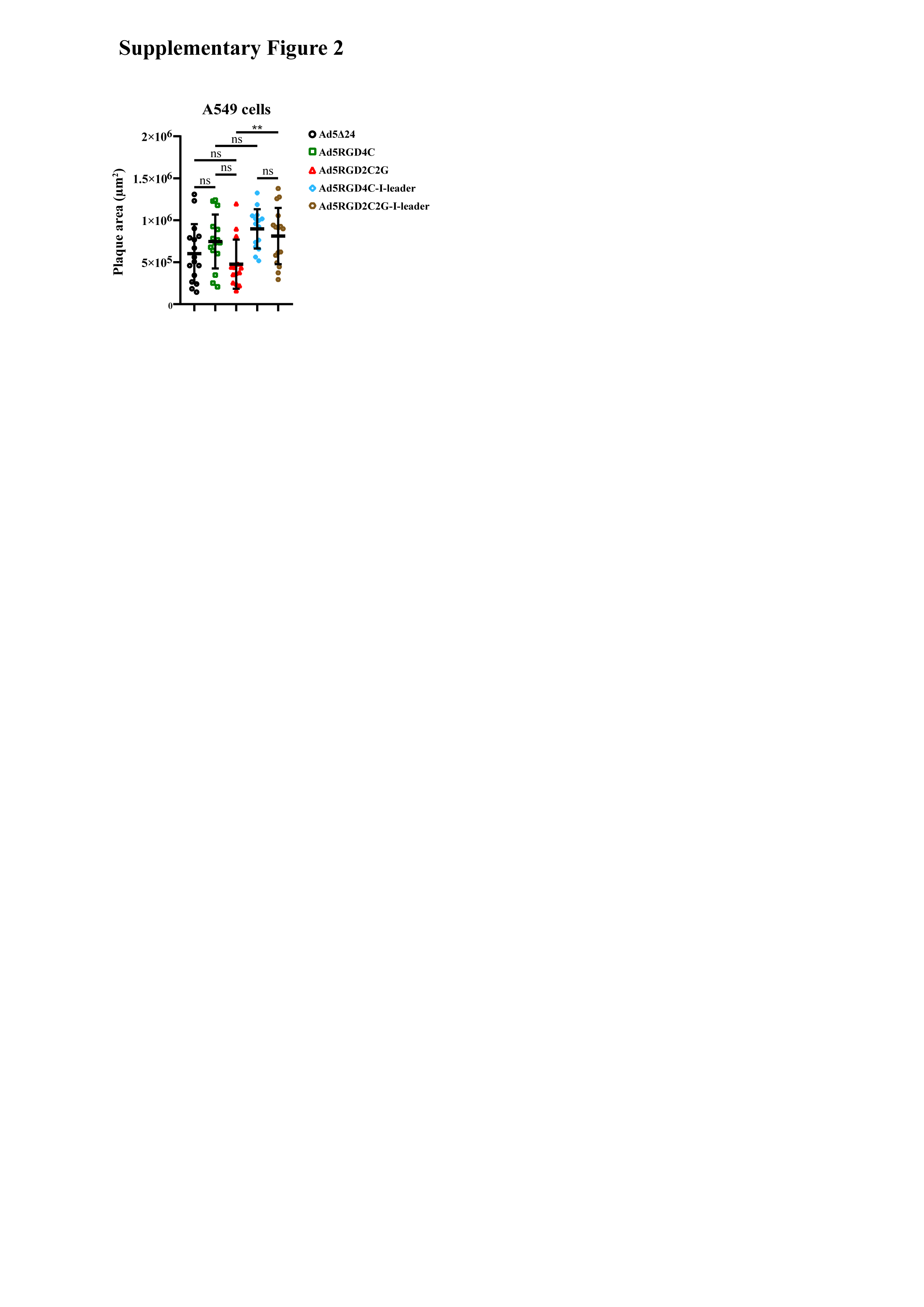


**Supplementary Figure 2.** Comparison of the mean plaque areas of the indicated recombinant adenoviruses in A549 cells at day 6 post-infection (1% agarose overlay). Data are presented as mean ±SD, **P < 0.01, by unpaired two-tailed t-test with Welch’s correction. The sample sizes are indicated in the figure (n=14-16).


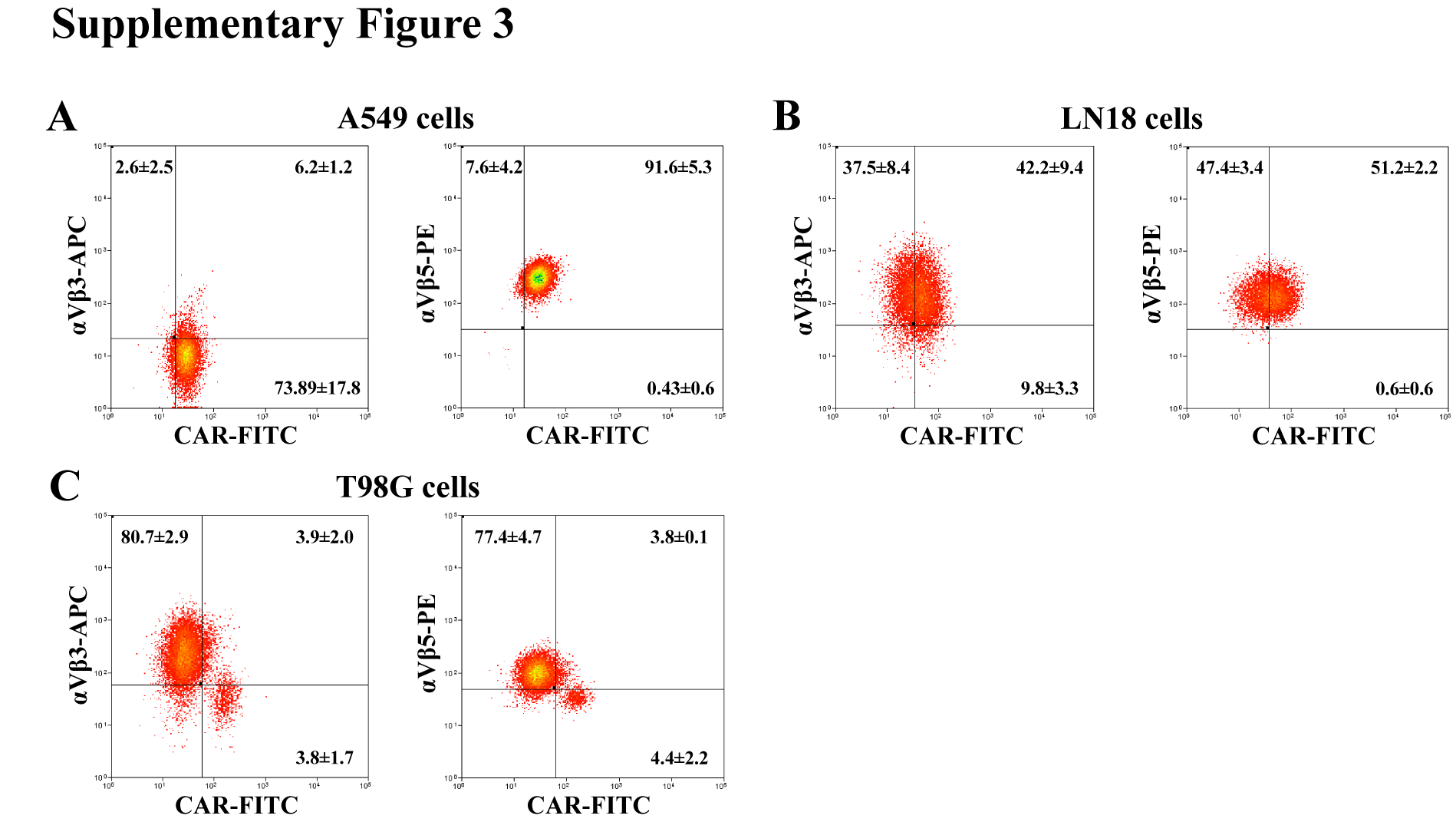


**Supplementary Figure 3.** Representative flow cytometry plots of expression of adenovirus 5 receptor (CAR) and co-receptors (αVβ3 and αVβ5 integrins) in human cell lines (T98G, LN18, and A549). The mean percentages ±SD of positive-stained cells from two independent experiments are shown.


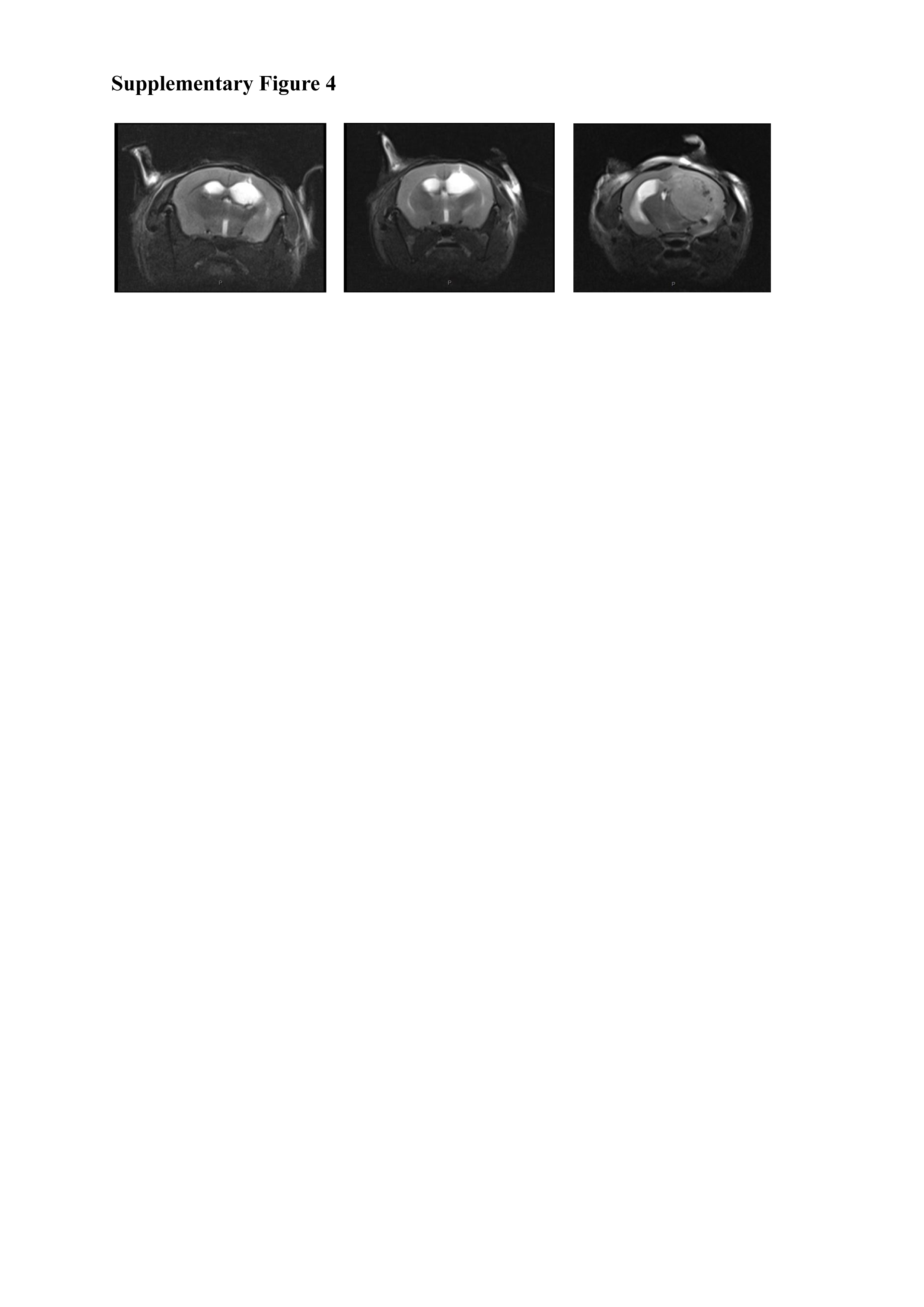


**Supplementary Figure 4.** The MRI scans of mice (left two pictures) that survived rechallenge with CT-2A glioma cells implanted into the contralateral hemisphere (left striatum). No tumor reappearance was observed in two out of three mice during >60 days of monitoring (n = 1, Ad5-delta-24-RGD4C; n=2, Ad5-delta-24-RGD2C2G). The MRI scan of a control animal (right picture) with large CT-2A tumor in the right hemisphere.

| **Supplementary Table 1. The oligonucleotides used for recombination (HPLC/PAGE-purified) and screening colony PCR/sequencing** | | |
| --- | --- | --- |
| **№** | **Oligonucleotide ID** | **Sequence** |
| **1** | Fwd_rec_rpsL-neo_E1Adelta24 | GAGAGCCTTGGGTCCGGTTTCTATGCCAAACCTTGTACCGGAGGTGATCGATGGCCTGGTGATGATGGC |
| **2** | Rev_rec_rpsL-neo_E1Adelta24 | CATAATCTAACACAAACTCCTCACCCTCTTCATCCTCGTCGTCACTGGGTGGTCAGAAGAACTCGTCAAGAAGG |
| **3** | Fwd_rec_E1Adelta24 | GAGAGCCTTGGGTCCGGTTTCTATGCCAAACCTTGTACCGGAGGTGATCGATCCACCCAGTGACGACGAGGATGAAGAGGGTGAGGAGTTTGTGTTAGATTATG |
| **4** | Rev_rec_E1Adelta24 | CATAATCTAACACAAACTCCTCACCCTCTTCATCCTCGTCGTCACTGGGTGGATCGATCACCTCCGGTACAAGGTTTGGCATAGAAACCGGACCCAAGGCTCTC |
| **5** | Fwd_rec_rpsL-neo_Fib5RGD | TAACACTAACCATTACACTAAACGGTACACAGGAAACAGGAGACACAACTGGCCTGGTGATGATGGC |
| **6** | Rev_rec_rpsL-neo_Fib5RGD | TAGTTGTGGCCAGACCAGTCCCATGAAAATGACATAGAGTATGCACTTGGTCAGAAGAACTCGTCAAGAAGG |
| **7** | Fwd_rec_Fib5RGD | TAACACTAACCATTACACTAAACGGTACACAGGAAACAGGAGACACAACTGCCTGTGACTGCCGCGGAGACTGTTTCTGCGGCCCAAGTGCATACTCTATGTCATTTTCATGGGACTGGTCTGGCCACAACTA |
| **8** | Rev_rec_Fib5RGD | TAGTTGTGGCCAGACCAGTCCCATGAAAATGACATAGAGTATGCACTTGGGCCGCAGAAACAGTCTCCGCGGCAGTCACAGGCAGTTGTGTCTCCTGTTTCCTGTGTACCGTTTAGTGTAATGGTTAGTGTTA |
| **9** | Fwd_rec_rpsL-neo_I-leader(125QStop) | TGTCCGCGCGCGGCGGTCGGAGCTTGATGACAACATCGCGGGCCTGGTGATGATGGC |
| **10** | Rev_rec_rpsL-neo_I-leader(125QStop) | TGACGCCGCGGGAGCTCCAGACCATGGACAGCTCCCATCTTCAGAAGAACTCGTCAAGAAGG |
| **11** | Fwd_rec_I-leader(125QStop) | ACCACGCCGCGCGAGCCCAAAGTCCAGATGTCCGCGCGCGGCGGTCGGAGCTTGATGACAACATCGCGTAGATGGGAGCTGTCCATGGTCTGGAGCTCCCGCGGCGTCAGGTCAGGCGGGAGCTCCTGCAGGTTTAC |
| **12** | Rev_rec_I-leader(125QStop) | GTAAACCTGCAGGAGCTCCCGCCTGACCTGACGCCGCGGGAGCTCCAGACCATGGACAGCTCCCATCTACGCGATGTTGTCATCAAGCTCCGACCGCCGCGCGCGGACATCTGGACTTTGGGCTCGCGCGGCGTGGT |
| **13** | Fwd_rec_rpsL-neo_Fib5/3 | CACAGGTGCCATTACAGTAGGAAACAAAAATAATGATAAGCTAACTTTGTGGACCGGCCTGGTGATGATGGC |
| **14** | Rev_rec_rpsL-neo_Fib5/3 | GAAATTTTCTGCAATTGAAAAATAAACACGTTGAAACATAACACAAACGATTCTTCAGAAGAACTCGTCAAGAAGG |
| **15** | Fwd_rec_Fib5/3 | CACAGGTGCCATTACAGTAGGAAACAAAAATAATGATAAGCTAACTTTGTGGACCGGTCCAAAACCAGAAGCC |
| **16** | Rev_rec_Fib5/3 | GAAATTTTCTGCAATTGAAAAATAAACACGTTGAAACATAACACAAACGATTCTTTATTTTAGTCATCTTCTCTAATATAGGAAAAGGT |
| **17** | Fwd_rec_Fib5/3RGD | CACAGGTGCCATTACAGTAGGAAACAAAAATAATGATAAGCTAACTTTGTGGACCGGTCCAAAACCAGAAGCC |
| **18** | Rev_rec_Fib5/3-RGD | GAAATTTTCTGCAATTGAAAAATAAACACGTTGAAACATAACACAAACGATTCTTTATTTTAGCCGCAGAAACAGTCTCC |
| **19** | Fwd_rec_rpsL-neo_Fib5/35 | TCTTACTCCTCCCTTTGTATCCCCCAATGGGTTTCAAGAGAGTCCCCCTGGGGTAGGCCTGGTGATGATGGC |
| **20** | Rev_rec_rpsL-neo_Fib5/35 | TGAAATTTTCTGCAATTGAAAAATAAACACGTTGAAACATAACACAAACGATTCTTCAGAAGAACTCGTCAAGAAGG |
| **21** | Fwd_rec_Fib5/35 | CTCCTCCCTTTGTATCCCCCAATGGGTTTCAAGAGAGTCCCCCTGGGGTACTTACTTTAAAATGTTTAACCCCAC |
| **22** | Rev_rec_Fib5/35 | TGCAATTGAAAAATAAACACGTTGAAACATAACACAAACGATTCTTTATTTTAGTTGTCGTCTTCTGTAATGTAAG |
| **23** | Fwd_rec_rpsL-neo_Fib5/35RGD | TCTTACTCCTCCCTTTGTATCCCCCAATGGGTTTCAAGAGAGTCCCCCTGGGGTAGGCCTGGTGATGATGGC |
| **24** | Rev_rec_rpsL-neo_Fib5/35RGD | TGAAATTTTCTGCAATTGAAAAATAAACACGTTGAAACATAACACAAACGATTCTTCAGAAGAACTCGTCAAGAAGG |
| **25** | Fwd_rec_Fib5/35RGD | CTCCTCCCTTTGTATCCCCCAATGGGTTTCAAGAGAGTCCCCCTGGGGTACTTACTTTAAAATGTTTAACCCCAC |
| **26** | Rev_rec_Fib5/35RGD | TGCAATTGAAAAATAAACACGTTGAAACATAACACAAACGATTCTTTATTTTAGCCGCAGAAACAGTCTCC |
| **27** | Fwd_rec_rpsl-neo_RGD2C2G | ACGGTACACAGGAAACAGGAGACACAACTGCCTGTGACTGCCGCGGAGACGGCCTGGTGATGATGGC |
| **28** | Rev_rec_rpsl-neo_RGD2C2G | TTGTGGCCAGACCAGTCCCATGAAAATGACATAGAGTATGCACTTGGGCCTCAGAAGAACTCGTCAAGAAGG |
| **29** | Fwd_rec_RGD2C2G | AACAGGAGACACAACTGCCTGTGACTGCCGCGGAGACGGATTCGGCCCAAGTGCATACTCTATGTCATTTTCATGGGACT |
| **30** | Rev_rec_RGD2C2G | AGTCCCATGAAAATGACATAGAGTATGCACTTGGGCCGAATCCGTCTCCGCGGCAGTCACAGGCAGTTGTGTCTCCTGTT |
| **31** | Fwd_seq_Ad5_E1Adelta24 | GAACCACCTACCCTTCACGA |
| **32** | Rev_seq_Ad5_E1Adelta24 | AACATGCCACAGGTCCTCAT |
| **33** | Fwd_seq(2)_Ad5_E1Adelta24 | CGACTCTGTAATGTTGGCGG |
| **34** | Rev_seq(2)_Ad5_E1Adelta24 | CAAACATGCCACAGGTCCTC |
| **35** | Fwd_seq_Fib5RGD | GCACAGCCTATACAAACGCT |
| **36** | Rev_seq_Ad5_Fib5RGD | CTATGTGGTGGTGGGGCTAT |
| **37** | Fwd_seq_RGD2C2G mut-spec | GCGGAGACGGATTCGGC |
| **38** | Rev_seq_RGD2C2G | CTATGTGGTGGTGGGGCTAT |
| **39** | Fwd_seq_Fib5/3 | GCCATAGCCATTAATGCAGGA |
| **40** | Rev_seq_Fib5/3 | TGTGTACTCTGTGTGTTGGGA |
| **41** | Fwd_seq_Fib5/3 mut-specific | CGCTGACTTTAGTGCAAGAGG |
| **42** | Fwd_seq_Fib5/35 | GGCTGCAAACTTTCTCCACA |
| **43** | Rev_seq_Fib5/35 | TGTGTACTCTGTGTGTTGGGA |
| **44** | Fwd_seq_Fib5/3RGD | TGCTTAATAAACGCCTGCCA |
| **45** | Rev_seq_Fib5/3RGD | GTTTGGCTCGACAGGAAACC |
| **46** | Fwd_seq_I-leader(125QStop) | GCAGAGTGGGAATTTGAGCC |
| **47** | Rev_seq_I-leader(125QStop) | CCTCTTGCAAGCCATCGAC |
| **48** | Fwd_seq_I-leader(125QStop) | GCTTGATGACAACATCGCGT |
| **49** | Fwd_seq_I-leader(125QStop) | GCTTGATGACAACATCGCCT |
| **50** | Rev_seq_I-leader(125QStop) | CCATGGACAGCTCCCATCAA |

| **Supplementary Table 2. The recombinant adenoviruses used in the cytotoxicity assays *in vitro* and in an orthotopic murine CT-2A glioma model** | | | | | |
| --- | --- | --- | --- | --- | --- |
| **№** | **Vector** | **VP/ml (×10^12)** | **IFU/ml (×10^11)** | **VP/IFU ratio** | **Polydispersity index (PDI)** |
| **1** | Ad5-delta-24 | 3,005 | 6,729 | 4.5:1 | NA |
| **2** | Ad5-delta-24-RGD2C2G (*in vitro* and *in vivo*) | 10,883 | 1,213 | 89:1 | 0,13 |
| **3** | Ad5-delta-24-RGD4C (*in vitro*) | 0,616 | 0,255 | 24:1 | 0,062 |
| **4** | Ad5-delta-24-RGD4C (*in vivo*) | 3,316 | 2,118 | 16:1 | 0,179 |
| **5** | Ad5-delta-24-RGD2C2G-I-leader(Q125Stop) | 4,543 | 1,724 | 26:1 | 0,093 |
| **6** | Ad5-delta-24-RGD4C-I-leader(Q125Stop) | 5,485 | 2,372 | 23:1 | 0,182 |

| **Supplementary Table 3. The number of adenoviral particles per cell required to produce 50% inhibition (IC50, vp/cell) in human and rodent cell lines** | | | | | | |
| --- | --- | --- | --- | --- | --- | --- |
| **Cell line** | **Days post-infection** | **Ad5-delta-24** | **Ad5-delta-24-RGD2C2G** | **Ad5-delta-24-RGD4C** | **Ad5-delta-24-RGD2C2G-I-leader(Q125Stop)** | **Ad5-delta-24-RGD4C-I-leader(Q125Stop)** |
| **T98G** | **7** | 6398 | 4973 | 1327 | 1148 | 607,6 |
| **C6** | **5** | 4999 | 5272 | 1615 | 5536 | 1881 |
| **CT26** | **5** | 16324 | 18323 | 5406 | 19196 | 6703 |
| **LN18** | **5** | NA | 282,4 | 193,9 | NA | NA |
| **CT-2A** | **5** | NA | 991,3 | 555,1 | NA | NA |
| **GL261** | **5** | NA | 3085 | 1480 | NA | NA |
